# Mammalian derived lipocalin and secretoglobin respiratory allergens strongly bind ligands with potentially immune modulating properties

**DOI:** 10.3389/falgy.2022.958711

**Published:** 2022-08-04

**Authors:** Bente Janssen-Weets, Frédéric Kerff, Kyra Swiontek, Stéphanie Kler, Rebecca Czolk, Dominique Revets, Annette Kuehn, Carsten Bindslev-Jensen, Markus Ollert, Christiane Hilger

**Affiliations:** ^1^Department of Infection and Immunity, Luxembourg Institute of Health, Esch-sur-Alzette, Luxembourg; ^2^Department of Dermatology and Allergy Center, Odense Research Center for Anaphylaxis, University of Southern Denmark, Odense, Denmark; ^3^Laboratory of Crystallography, Center for Protein Engineering-InBioS, University of Liège, Liège, Belgium; ^4^Faculty of Science, Technology and Medicine, University of Luxembourg, Esch-sur-Alzette, Luxembourg

**Keywords:** lipocalin, secretoglobin, mammalian respiratory allergens, protein-ligand interactions, fluorescence-quenching assays, farnesol, Cav p 1 crystal structure

## Abstract

Allergens from furry animals frequently cause sensitization and respiratory allergic diseases. Most relevant mammalian respiratory allergens belong either to the protein family of lipocalins or secretoglobins. Their mechanism of sensitization remains largely unresolved. Mammalian lipocalin and secretoglobin allergens are associated with a function in chemical communication that involves abundant secretion into the environment, high stability and the ability to transport small volatile compounds. These properties are likely to contribute concomitantly to their allergenic potential. In this study, we aim to further elucidate the physiological function of lipocalin and secretoglobin allergens and link it to their sensitizing capacity, by analyzing their ligand-binding characteristics. We produced eight major mammalian respiratory allergens from four pet species in *E.coli* and compared their ligand-binding affinities to forty-nine ligands of different chemical classes by using a fluorescence-quenching assay. Furthermore, we solved the crystal-structure of the major guinea pig allergen Cav p 1, a typical lipocalin. Recombinant lipocalin and secretoglobin allergens are of high thermal stability with melting temperatures ranging from 65 to 90°C and strongly bind ligands with dissociation constants in the low micromolar range, particularly fatty acids, fatty alcohols and the terpene alcohol farnesol, that are associated with potential semiochemical and/or immune-modulating functions. Through the systematic screening of respiratory mammalian lipocalin and secretoglobin allergens with a large panel of potential ligands, we observed that total amino acid composition, as well as cavity shape and volume direct affinities to ligands of different chemical classes. Therefore, we were able to categorize lipocalin allergens over their ligand-binding profile into three sub-groups of a lipocalin clade that is associated with functions in chemical communication, thus strengthening the function of major mammalian respiratory allergens as semiochemical carriers. The promiscuous binding capability of hydrophobic ligands from environmental sources warrants further investigation regarding their impact on a molecule's allergenicity.

## Introduction

Sensitization to furry animals constitutes a risk factor for the development of IgE-mediated allergic diseases such as rhinitis, conjunctivitis or asthma (1). Sensitization rates lie between 10 and 12% in the general population in Europe and the United States (2, 3). Allergic sensitization and clinical allergy not only result from direct contact (e.g., contact with pets), but also through indirect exposure as mammalian respiratory allergens are perennial and ubiquitous. They become easily airborne and are transported into public areas, most likely by sticking to human hair or clothing (4). Furry mammals, such as dogs, cats, rabbits and rodents, are a common source of respiratory allergens in households and occupational areas.

Among mammalian respiratory allergens, the most prominent protein family is the lipocalin family. More than twenty lipocalins from different animal species are known allergens. Mostly, they are major allergens with specific IgE (sIgE) prevalence rates over 50% in sensitized individuals (5). Dog dander allergy is associated with complex sIgE-profiles, particularily multiple sensitizations to several dog lipocalins (6). Indeed, co-sensitization to several mammalian lipocalin allergens has been associated with severe asthma (7). On the contrary, the protein family of secretoglobins only comprises two characterized allergens to date: Fel d 1 and Ory c 3. Fel d 1 is by far the most relevant cat allergen and a good marker for cat allergy, with a sensitization rate over 90% (8, 9). The Fel d 1-homolog Ory c 3, a major allergen from rabbit dander, was detected in settled household dust and reported to have a high sensitizing rate (77%) in rabbit allergic patients (10). Though lipocalin and secretoglobin allergens possess different molecular features, both share common traits. They are both small secretory proteins that are commonly found in fur and bodily fluids, such as urine, saliva, sweat and sebum (4).

Lipocalins are a highly diverse protein family and on average, its members (allergens and non-allergens) share <30% amino acid sequence identity. They all feature highly conserved β-barrel structures that enable them to transport small volatile hydrophobic compounds. Some lipocalins are highly conserved throughout evolution and specialized in their essential functions, such as retinol-binding proteins (RBPs) (11), whereas others seem to have multiple functions. One example is the human lipocalin-1 (LCN1). LCN1 is a promiscuous binding protein that can modulate the tear film viscosity, but also acts as a scavenger of antimicrobial lipids (12).

Secretoglobins, formerly known as uteroglobins, are dimeric alpha-helical proteins that also bind hydrophobic ligands (13, 14). In humans, they are mostly studied in the context of immunoregulatory processes of airway diseases (15). For example Secretoglobin family 1A member 1 (SCGB1A1) is highly abundant in lining fluids of the respiratory tract and has anti-inflammatory properties (15). In mammalian animals however, lipocalins and secretoglobins are mostly noted in their function as semiochemicals or semiochemical carriers. These molecules direct a variety of social behaviors, for example alarm signaling, territory marking, mating and maternal behavior (16–20). Lipocalins and secretoglobins are abundantly secreted into the environment and are the most prevalent proteins found in nesting material of mice (21). Their high stability allows a persistent spreading and enhances the slow release and longevity of their chemical message (22–24). These factors, abundancy and stability, are also hallmarks of many allergenic molecules and may partly explain why some members of both protein families are allergens (25).

In general, mammalian respiratory allergens are understudied concerning their physiological function. Based on similarities in their amino acid sequence identity to semiochemical carriers, their active secretion into the environment and their ability to bind and release small volatile compounds, a role in chemical communication is assumed. Only few respiratory allergens have been subject to isolated *in vitro* and *in silico* ligand-binding studies before (26–31). Results confirm the ability to bind potent pheromones from different chemical classes, such as steroids (27), thiazolines (29, 31), sesquiterpenes (30), fatty acids (27) and fatty alcohols (26). The major horse allergen Equ c 1 was purified from horse sweat together with its endogenous ligand oleamide (26) but its function is not clear. Whereas, oleamide is the only natural ligand that has been characterized for furry animal allergens, more information is available on ligands of plant non-specific lipid transfer proteins and the family 10 of pathogenesis-related proteins (32). Ligands binding to allergenic proteins can alter characteristics that contribute to their sensitizing capacity, or can have intrinsic immunomodulatory capacities themselves (32). The identification of preferred structures that strongly bind to allergenic transport proteins might not only give insight into their physiological function, but could serve as a complementing factor in future investigations by mimicking a more natural state of secreted, allergenic transport proteins.

We report here the first comparative ligand-binding study of 8 major mammalian respiratory allergens from 4 pet species. We systematically screened a large panel of molecules involved in chemical communication and immunoregulation, some of their derivatives, as well as bacterial compounds and analyzed their binding affinities to these 8 allergens and human LCN-1. Furthermore, we solved the crystal structure of the major guinea pig allergen Cav p 1, a lipocalin closely related to the well-known semiochemical hamster aphrodisin, allowing us to compare the binding cavities of mammalian respiratory allergens belonging to three different lipocalin subgroups.

## Materials and methods

### Production, quality control and secondary structure analysis of recombinant allergens

Recombinant proteins were produced in *Escherichia coli* as previously described for Ory c 3 [UniProtKB accession number (AC): Q9GK63 (chain 1) + Q9GK67 (chain 2)], Cav p 1.0101 (AC: A0A484HM70) and Cav p 1.0201 (AC: A0A484HRI4) (33), Fel d 4 (AC: Q5VFH6) and Can f 6 (AC: H2B3G5) (34), and Fel d 1 [AC: P30438 (chain 1) + P30440 (chain 2)] (35). Synthetic cDNA sequences coding for the mature proteins Can f 1 (AC: O18873), LCN1 (AC: P31025) and Ory c 2 (GenBank AC: ON098933) followed by a 6 histidine tag were cloned into vector pEX-A128 by Eurofins (Munich, Germany) and subcloned into vector pET-21d(+) for expression in the *E.coli* strain Rosetta-gami™ 2(DE3) (Novagen^®^ by Merck, Darmstadt, Germany) and purified as described (33). Briefly, the recombinant proteins were purified by immobilized metal ion affinity chromatography (HisTrap HP, GE Healthcare, Buckinghamshire, UK) under native conditions followed by anion exchange using a Resource Q column (GE Healthcare) and a linear gradient of 0–500 mM NaCl in 20 mM TRIS-HCl, pH 7.2. The recombinant protein solutions were dialyzed against phosphate buffered saline (PBS) and concentrated to 1–2 mg/mL.

The purity of the recombinant proteins was analyzed by SDS-PAGE under reducing conditions followed by silver staining (Pierce™ Silver Stain Kit, Thermo Fisher Scientific Inc.). Intact mass of recombinant proteins was precisely measured by Matrix Assisted Laser Desorption/Ionization time-of-flight mass spectrometry (MALDI-TOF) in linear mode. Recombinant proteins were treated with dithiothreitol to reduce disulfide bridges and then they were digested with trypsin. The Protein Mass Fingerprints (PMFs) acquired in reflectron mode on the MALDI-TOF were compared with MASCOT 2.0 search engine (Matrix Science, UK) in the NCBInr protein database to prove their sequence identity. All mass spectra were acquired on a MALDI TOF/TOF instrument (Ultraflex I; Bruker Daltonics, Bremen, Germany) as previously described (10).

Secondary protein structure formation and thermal stability of the recombinant proteins were determined by circular dichroism (CD) spectroscopy. Recombinant proteins were diluted with ultrapure water to a concentration of 10 μmol/L in a volume of 300 μL to measure their CD spectra using the Chirascan CD spectrometer (Applied Photophysics). Far-ultraviolet CD spectra were recorded at 180–260 nm (0.8 nm bandwidth, 0.4 s interval, 3 repeats) in a cuvette of 0.1 cm path length. Thermal sensitivity was assessed by ramping temperatures (20–95°C) with an internal temperature probe and continuous CD-signal measurements (ramping rate 0.82°C per minute, no repeats). After 10 min at 95°C another CD spectrum was recorded with 3 repeats, then the temperature was decreased back to 20°C for a final scan with 3 repeats to analyze the refolding capacity. The read-out was converted with respective protein details, such as molecular weight and number of amino acids, into degrees^*^cm2^*^dmol−1. Chirascan Global 3 Analysis Software (Applied Photophysics) and DichroWeb online server (36) with solutions from the CDSSTR method and reference data set 7 (37) were used to analyze and interpret CD spectra, measured as a function of temperature.

### Analysis of IgE-binding to recombinant and native proteins by ELISA

IgE-binding of recombinant allergens was compared to their native form by ELISA using sera of patients allergic to furry animals. Patients had been recruited at the National Unit of Immunology- Allergology at the Centre Hospitalier de Luxembourg in the frame of previous studies (10, 34, 38). Ethical approval was obtained from the National Committee for Medical Research Ethics (CNER 2010 01/06 and 2013 07/04) and informed consent was obtained for all subjects. Quantification of specific IgE (sIgE) to cat (e1; 14 sera, range: 3 to >100 kU_A_/L), dog (e5; 13 sera, range: 18 to >100 kU_A_/L), guinea pig (e6; 12 sera, range: 12 to >100 kU_A_/L) and rabbit (e82; 10 sera, range: 24 to >100 kU_A_/L) epithelium and/or dander was performed with ImmunoCAP (ThermoFisher Scientific Inc., Uppsala, Sweden).

Native allergens nCan f 1 and nFel d 1 were purchased (Indoor Biotechnologies, Inc., Charlottesville, VA). Native nCav p 1 was isolated from the harderian gland of the guinea pig as a pool containing both isoallergens nCav p 1.0101 and nCav p 1.0201 (39), native nOry c 3 and nOry c 2 were isolated from rabbit hair extracts (10). Native Can f 6 was isolated from a dog dander extract (Allergon, Ängelholm, Sweden) that was solved in 20 mM TRIS-HCl, pH 8, centrifuged and filtered through a 0.22 μM PVDF membrane (Millipore; Merck, Darmstadt, Germany). Proteins were separated by 3-step anion exchange chromatography. A HiTrap Capto DEAE column (GE Healthcare) was used followed by a Resource Q column (GE Healthcare). Bound proteins were eluted by using a linear gradient of 0–500 mM NaCl in 20 mM Bis-TRIS, pH 7. Eluted fractions were analyzed by SDS-PAGE followed by silver staining, than by immunoblotting with polyclonal anti-Can f 6 mouse serum as described (34). Individual fractions containing proteins recognized by the anti-Can f 6 mouse serum were selected for N-terminal sequencing. Fractions containing nCan f 6 were pooled and further purified by a third ion exchange chromatography step using a Mono Q column (GE Healthcare) with a linear gradient of 0–500 mM NaCl in 20 mM Bis-TRIS, pH 6.3. The nCan f 6 pool was analyzed by SDS-PAGE under reducing conditions and stained with GelCode™ Blue (Thermo Fisher Scientific Inc.). The purity of the nCan f 6 pool is estimated to be 80%. sIgE to native and recombinant allergens were quantified by ELISA as previously described (40). The proteins were coated at a concentration of 5 μg/mL in PBS. Patient sera were diluted 5–10-fold in 3% BSA in PBS. A pool of 22 sera from patients allergic to mites and/or pollen were used as negative controls.

### Crystallization and structure determination of Cav p 1.0101

Cav p 1.0101 was concentrated to 4 mg/mL in Tris-HCl 20 mM pH 7 and crystallized using the sitting-drop vapor diffusion method. 0.25 μL of protein was mixed with 0.25 μL of precipitant solution (polyvinylpyrrolidone K 15 50% w/v, citrate 0.1 M pH 5), 0.05 μL of 6-Aminohexanoic acid 30% w/v and 0.05 μL of 1-decanol 1 mM in ethanol 1% v/v.

Crystals grew at room temperature. The crystals were transferred into a cryoprotectant solution containing glycerol 45% v/v and polyethylene glycol 6,000 45% w/v before flash-freezing in a liquid nitrogen bath. Diffraction data were collected at the Soleil Synchrotron Proxima 1 beamline (Saint-Aubin, France). Data were integrated and scaled using XDS (41). Initial phases were obtained by molecular replacement using the structure of aphrodisin, a sex pheromone from female hamster as a search model (PDB code 1E5P, https://doi.org/10.1006/jmbi.2000.4241) using Phaser (42). The A molecule was built with Coot (Crystallographic object-oriented toolkit) (43) and molecules B, C, D, and E were obtained by applying the non-crystallographic symmetry (ncs). The refinement was done with BUSTER refine using automatically generated ncs restraints (44). The figures were prepared using PyMOL (The PyMOL Molecular Graphics System, Version 2.5.2 Enhanced for Mac OS X, Schrödinger, LLC). The structure was deposited in the Protein Data Bank and received the code 8A0D.

### Fluorescence quenching assays

Fluorescence quenching assays were performed as previously described (45), with modifications. Fluorochromes and ligands were purchased from Sigma Aldrich (Steinheim, Germany), Carl Roth (Karlsruhe, Germany) and TCI Europe N.V. (Zwijndrecht, Belgium). Tested ligands were arachidonic acid; cholesterol; cyclohexene; decanoic acid; 1-decanol; dihydroxytestosterone; 3,7-dimethyl-1-octanol; dimethylsulfide; dodecanoic acid; 1-dodecanol; eicosanoic acid; 1-eicosanol; 4-ethylphenol; farnesol; geranyl nitrile; hexadecanoic acid; 1-hexadecanol; isoamyl acetate; 2-isobutyl-3-methoxypyrazine (IBMP); 2-isopropylphenol; 3-ilsopropylphenole; 4-isopropylphenol; linalool; methyl hexadecanoate; methyl octadecanoate; methyl thiobutyrate; 2-naphthol; octadecanoic acid; 1-octadecanol; Z-9-octadecen-1-ol/oleyl alcohol; 1-octanol; oleamide; 1-octanal; octanamine; pentadecanoic acid; pentadecanol; 1-pentene; 2-phenylethanol; progesterone; 4-propylphenol; pyrocatechol; retinol; testosterone; tetradecanoic acid; tetradecanol; tyramine; ammonium iron (III) citrat; lipopolysaccharide (LPS) (Escherichia coli O111:B4); monophosphoryl lipid A (MPLA).

Stock solutions were prepared at a concentration of 10 mM in 100% ethanol for the fluorochromes 1-aminoanthracene (1-AMA), 8-anilino-1-naphthalensulfonic acid (1,8-ANS) and for all ligands, and in 100% methanol for n-phenyl-1-naphthylamine (1-NPN), respectively. Iron catecholate ligands (siderophores) were prepared as previously described (46). Fluorochrome-protein complexes were analyzed at an equimolar concentration of 5 μM in PBS with 5% ethanol in a volume of 100 μL per well in black 96-well non-binding microplates (Greiner Bio-One GmbH, Frickenhausen, Germany). Solutions containing only protein or the respective fluorescent probe in PBS with 5% ethanol served as controls. Fluorochrome-protein complexes were incubated with orbital shaking for 10 min at 500 rpm at room temperature (RT) before measurement using the fluorescence reader TECAN Infinite 200 Pro (Tecan Trading AG, Switzerland). Excitation wavelengths were fixed at 380 nm for 1-AMA and 1,8-ANS, and 337 nm for 1-NPN. Fluorescence emission spectra were recorded in 5 nm steps between 420 and 560 nm for 1-AMA and 1,8-ANS and between 380 and 500 nm for 1-NPN. The establishment of fluorescent quenching assay parameters for each allergen is explained in the results Section Fluorescence-quenching assay set up. Binding affinities were analyzed by non-linear regression in GraphPad Prism Version 9.3.1 (GraphPad Software, LLC). For the ligand competition assays, ligands were added to fluorochrome-allergen complexes with a final buffer composition of 5% ethanol in PBS in a volume of 100 μL and incubated for 30 min at 500 rpm at RT and further equilibrated for another 30 min without shaking before fluorescence measurement.

### Statistical and structural analysis and data visualization tools

Statistical analysis and graph design were performed with GraphPad Prism Version 9.3.1 (GraphPad Software, LLC). Heatmap analysis was generated with RStudio Version 2022.02.0+443 “Prairie Trillium” including the packages tidyverse (47) and cowplot (48). Prinicpal component analysis (PCA) was created with the packages FactoMineR (49), factoextra (50) and corrplot (51). Clustal Omega (52) was used for multiple sequence alignments and for the creation of phylogenetic trees by neighbor joining.

*In silico* structure modeling of proteins was performed using AlphaFold2 (53). Structures were analyzed with CASTp 3.0 (54) and PISA (55). Chemical structures were created with ChemDraw JS (PerkinElmer Informatics).

## Results

### Recombinant allergens are of high quality in terms of purity and IgE-binding

We selected major respiratory allergens derived from common furry pets to investigate their ligand-binding properties. In total, we chose eight allergens derived from four pet species: dog (Can f 1, Can f 6), cat (Fel d 1, Fel d 4), rabbit (Ory c 2, Ory c 3) and guinea pig (Cav p 1.0101, Cav p 1.0201). Six of the allergens are lipocalins, whereas Fel d 1 and Ory c 3 are members of the secretoglobin family. Lipocalin-1 (LCN1), also known as tear lipocalin or von Ebner's gland (VEG) protein, was used as a control for a human homologous non-allergenic lipocalin.

**Figure 1:**
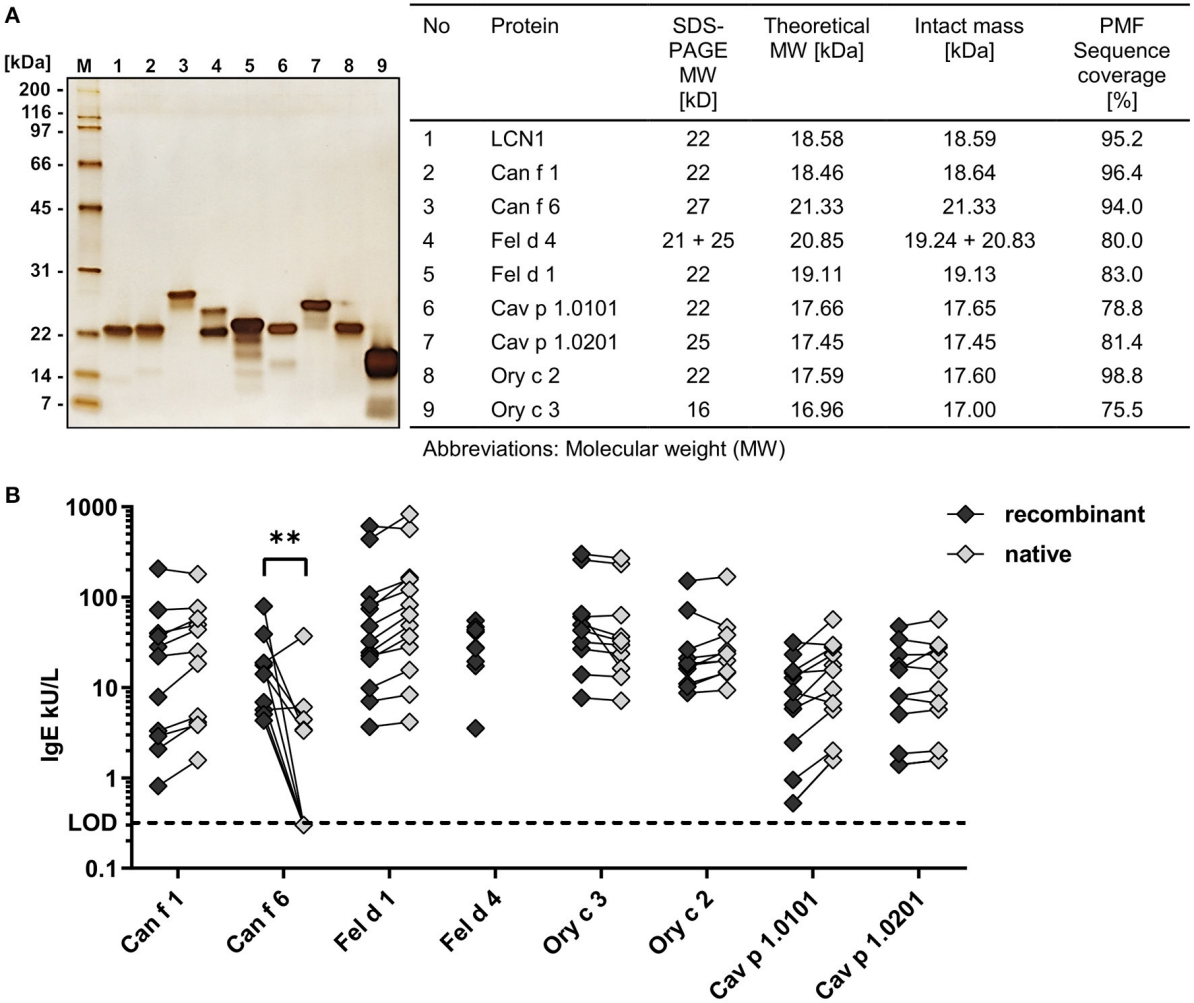
Recombinant allergens are of high purity and show a comparable IgE-binding to their native counterpart. **(A)** SDS-PAGE and silver stain of eight purified recombinant mammalian respiratory allergens and the human homolog LCN1 (200 ng protein per well). Intact masses and amino acid sequence identity of recombinant allergens and LCN1 were analyzed by mass spectrometry. **(B)** Comparison of specific IgE-binding of allergic patient sera to recombinant allergens and their native counterpart by ELISA (*n* = 9–13 patients). Median IgE-reactivity was compared between the recombinant and the native form by Multiple Mann-Whitney test (^**^P-value = 0.0028). Statistical analysis using Wilcoxon matched-pairs signed rank test between recombinant and native allergens resulted in no discoveries [FDR (Q) = 1%]. No native Fel d 4 was available. Lower limit of detection (LOD) ≤ 0.32 kU/L.

Allergens were produced as recombinant proteins containing a C-terminal polyhistidine tag in the *E. coli* strain Rosetta-gami 2 to enhance disulphide bond formation. Allergens were purified by immobilized metal ion affinity chromatography with subsequent anion exchange chromatography. This 2-step approach resulted in allergen preparations of high purity as confirmed by SDS-PAGE followed by silver staining and mass spectrometry (Figure 1A). Allergen bands appeared between 16 and 27 kDa. Allergens formed single bands except for cat lipocalin Fel d 4 showing a clear double band pattern that matched the mass spectrometry results. Peptide mass finger printing (PMF) of cat secretoglobin Fel d 1 revealed progressive C-terminal degradation, which was also seen during SDS-PAGE appearing as faint bands in the lower masses.

For most allergens, native molecules were available and IgE-binding of the recombinant allergens was compared to the IgE-binding of native allergens, using sera from patients sensitized to cat, dog, guinea-pig or rabbit. Recombinant allergens showed a similar IgE-reactivity compared to their native counterparts in ELISA measurements, indicating the presence of well-structured allergens with functional IgE-epitopes (Figure 1B). All native allergens were of high purity, except for native Can f 6 (nCan f 6), which contained ~20% of impurities (data not shown). The significantly lower IgE-reactivity from five out of ten patient sera to nCan f 6 compared to the recombinant form could be explained by the lower nCan f 6 content caused by the impurities in the nCan f 6 pool. Native Cav p 1 was only available as a mixture of both isoallergens, nCav p 1.0101 and nCav p 1.0201.

We confirmed the identity, purity and IgE-binding of our recombinant allergens, thus providing the source material of highest quality for analyzing the structure and ligand binding properties of mammalian respiratory allergens in fluorescence quenching assays.

### Crystallographic structure of Cav p 1.0101

**Figure 2:**
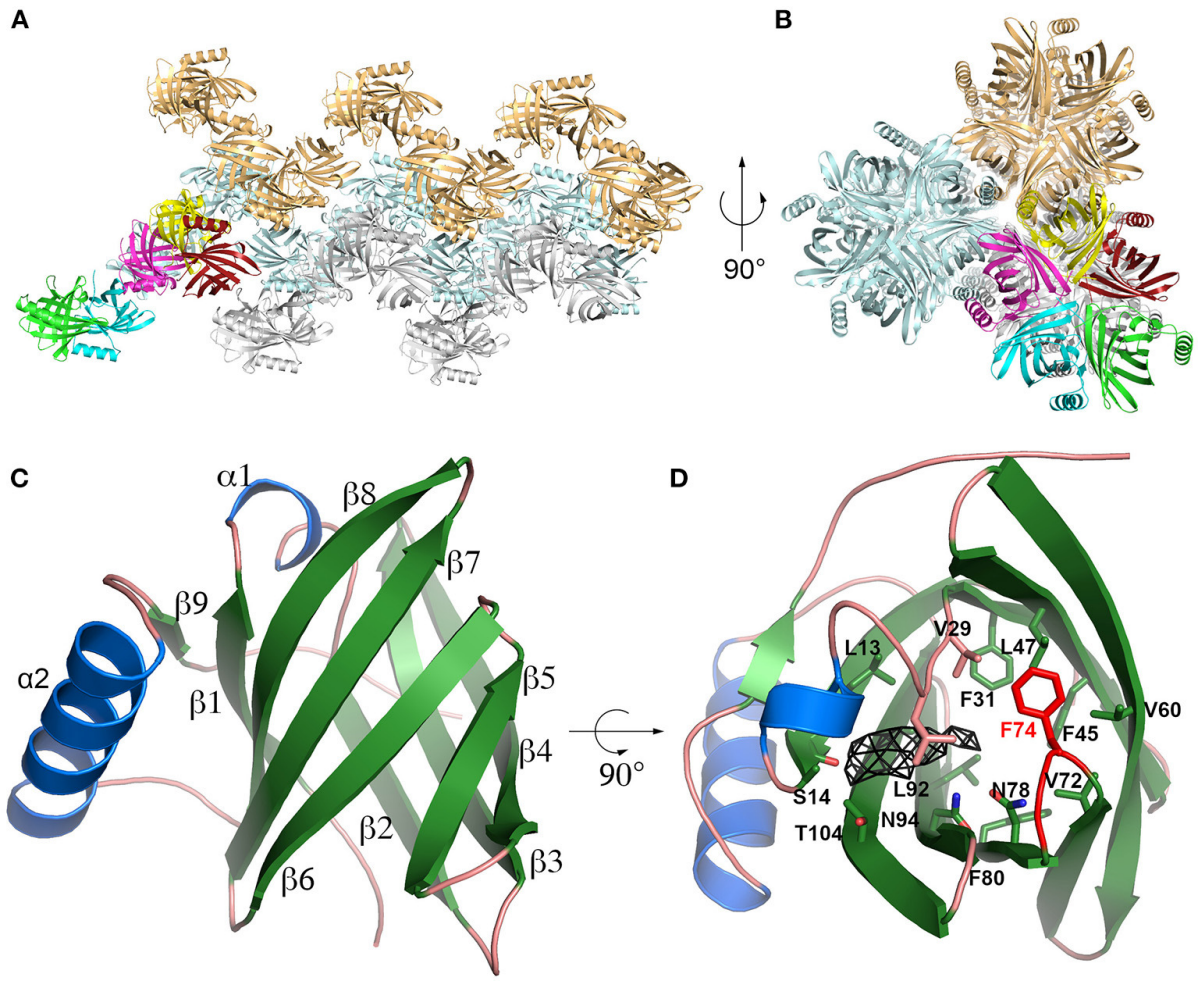
Cav p 1.0101 crystal structure. **(A,B)** Cartoon representation of Cav p 1.0101 in two perpendicular orientations highlighting the stacking of helices observed in the crystal, with the five monomers of one asymmetric unit in bright colors. **(C)** Overall fold of Cav p 1.0101 with α-helix in blue and β-strands in green. **(D)** Same representation as **(C)** with residues defining the central cavity shown as sticks, the β5β6 loop is in red, and the electron density (Fo-Fc map at 2 sigma level) observed in central cavity of monomer E as a black mesh.

The existence of two closely related Cav p 1 isoallergens (amino acid identity 82.5%) in guinea-pig and the fact that Cav p 1.0201 is a specific marker of allergy to guinea-pig with an amino acid identity to other known animal allergens below 47% (39), prompted us to determine the structure of the isoallergens. We could solve the crystallographic structure of Cav p 1.0101 at 3.7 Å. The crystal belongs to the P2_1_2_1_2_1_ space group with five molecules in the asymmetric unit. In the crystal, these molecules are organized as a stacking of helical superstructure with five molecules per turn along the a-axis (Figures 2A,B). The Cav p 1.0101 structure is characterized by R_work_ and R_free_ values of 25.6 and 26.2%, respectively (Supplementary Table 1) and contains residues 4 to 150. The first three residues as well as the last seven, which include the His_6_-Tag could not be built because of a lack of electron density. The interface between monomers covers an average area of 654 Å^2^ that is not considered stable in solution according to PISA (55).

Cav p 1.0101 adopts a classic lipocalin fold: an eight stranded β-barrel (residues 9-112) with a short helix insertion between β1 and β2, followed by a 14 residue long α-helix and a short β-strand forming antiparallel interactions with β1 (Figure 2C). The five monomers in the asymmetric unit were refined using non-crystallographic symmetry restrains and display a maximum root mean square deviation (rmsd) of 0.142 Å between monomers, calculated over the 147 Cα.

The crystal used to obtain the structure of Cav p 1.0101 was obtained in the presence of 1-decanol. An electron density that is observed in the central cavity of monomer E (Figure 2D), likely indicates the presence of a 1-decanol molecule. In the four other monomers, the corresponding density is significantly weaker. This density is adjacent to the carbonyl oxygen of Ser14, one of the very few polar atoms on the surface of the cavity available for hydrogen bonding, which is likely involved in an H-bond with the hydroxyl group of 1-decanol. However, our attempt to model 1-decanol led to an unrealistic conformation of its aliphatic chain due to a poor electron density for this region. This phenomenon likely results from the flexibility and the multiple conformations that this aliphatic chain can adopt in the cavity.

The Cav p 1.0101 cavity does not display any access to the solvent. The β-barrel side open to the solvent in the LCN1 structure is occupied by the sidechain of Phe74, which belongs to the short β5β6 loop in Cav p 1.0101. This loop, which is part of the interface between monomers in the crystal likely acts as a lid capable of opening for the ligands to access the central cavity (Figure 2D).

### Mammalian respiratory allergens are highly resistant to thermal denaturation

**Figure 3:**
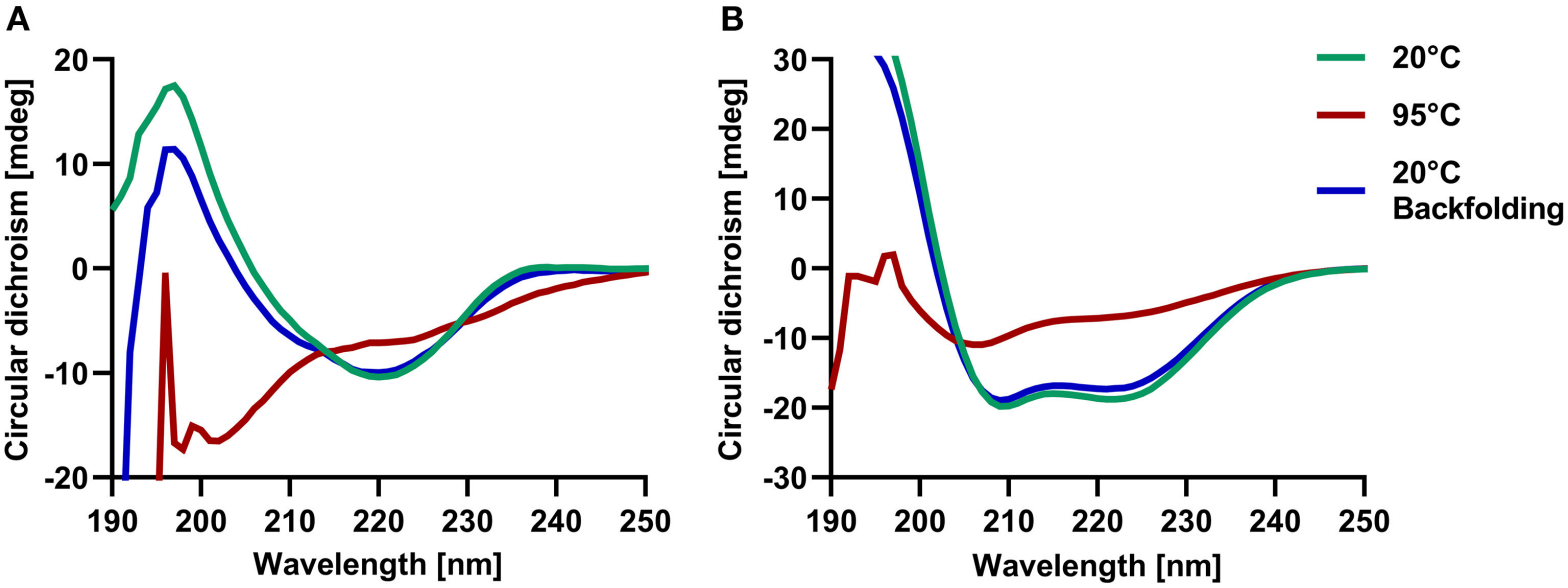
Circular dichroism spectroscopy confirmed the beta-sheet and alpha-helical structures of respective lipocalins and secretoglobins and revealed a high thermal stability of allergens. Examples of circular dichroism spectra at 20°C (green) and after temperature ramping at 95°C (red) and 20°C (blue) of guinea pig lipocalin Cav p 1.0101 **(A)** and rabbit secretoglobin Ory c 3 **(B)**.

In order to assess the correct folding of the recombinant molecules and to determine their thermal stability, we further analyzed them by circular dichroism (CD) spectroscopy. Lipocalins exhibited a typical CD curve that reflects a high beta-sheet content with a maximum between 191 and 197 nm and a minimum between 208 and 220 nm. Secretoglobins were characterized by curves that are typical for alpha-helical structures with two distinct minima at 209 nm and 221/222 nm, as well as a maximum at 191/193 nm (Figure 3; Table 1). Temperature ramping up to 95°C revealed that both lipocalin and secretoglobin allergens are highly stable proteins that are able to fold back into their original shape after heat treatment. Allergens with higher melting points showed a greater capacity to refold. Melting points ranged from 66 to 86°C for lipocalins and from 88 to 90°C for secretoglobins, confirming a very high thermal stability and refolding capacity of all molecules.

**Table 1:** Melting points and characteristic maxima and minima for recombinant lipocalin and secretoglobin allergens and human LCN1 analyzed by circular dichroism spectroscopy combined with temperature ramping.

**Protein**	**Melting point**	**Maximum**		**1st minimum**		**2nd minimum**	
	**(C°)**	**Wavelength (nm)**		**Wavelength (nm)**		**Wavelength (nm)**	
		**20°C**	**Back-folding**	**20°C**	**Back-folding**	**20°C**	**Back-folding**
**Lipocalins**							
LCN1	74.8 ± 0.2	192	191	208	203	–	–
Can f 1	85.9 ± 0.6	195	191	208	208	–	–
Can f 6	65.9 ± 0.1	199	194	219	211	–	–
Fel d 4	65.6 ± 0.1	199	197	217	206	–	–
Cav p 1.0101	71.0 ± 0.1	197	197	220	219	–	–
Cav p 1.0201	71.0 ± 0.1	197	194	219	217	–	–
Ory c 2	74.1 ± 0.1	195	194	218	218	–	–
**Secretoglobins**							
Ory c 3	87.6 ± 0.2	191	191	209	209	221	221
Fel d 1	90.1 ± 0.5	193	193	209	209	222	222

### Fluorescence-quenching assay set up

Lipocalins form a hydrophobic internal cavity as part of their function as carrier proteins for small hydrophobic compounds in an aqueous environment. Secretoglobins form hydrophobic cavities as well, but their function as transport proteins needs further investigation. Fluorescence quenching assays are based on the binding of fluorescent probes within this hydrophobic region, which is accompanied by a blue shift in emission spectra and a significant increase in fluorescence intensity. Fluorochromes such as 1-AMA, 1,8-ANS and 1-NPN have been used successfully in combination with lipocalin and secretoglobin proteins in fluorescence quenching assays before (26, 27, 56, 57), assuming a binding stoichiometry of fluorochrome and protein of 1:1.

We established four specific parameters for each allergen to set up a fluorescence quenching assay for screening a large array of potential ligands: (i) a compatible fluorochrome, (ii) the optimal emission wavelength to analyze the fluorescence intensities of the fluorochrome-allergen complex, (iii) the binding affinities of the fluorochrome-allergen complex (K_d_ values) and (iv) optimal fluorochrome concentrations (EC_50_ values).

**Figure 4:**
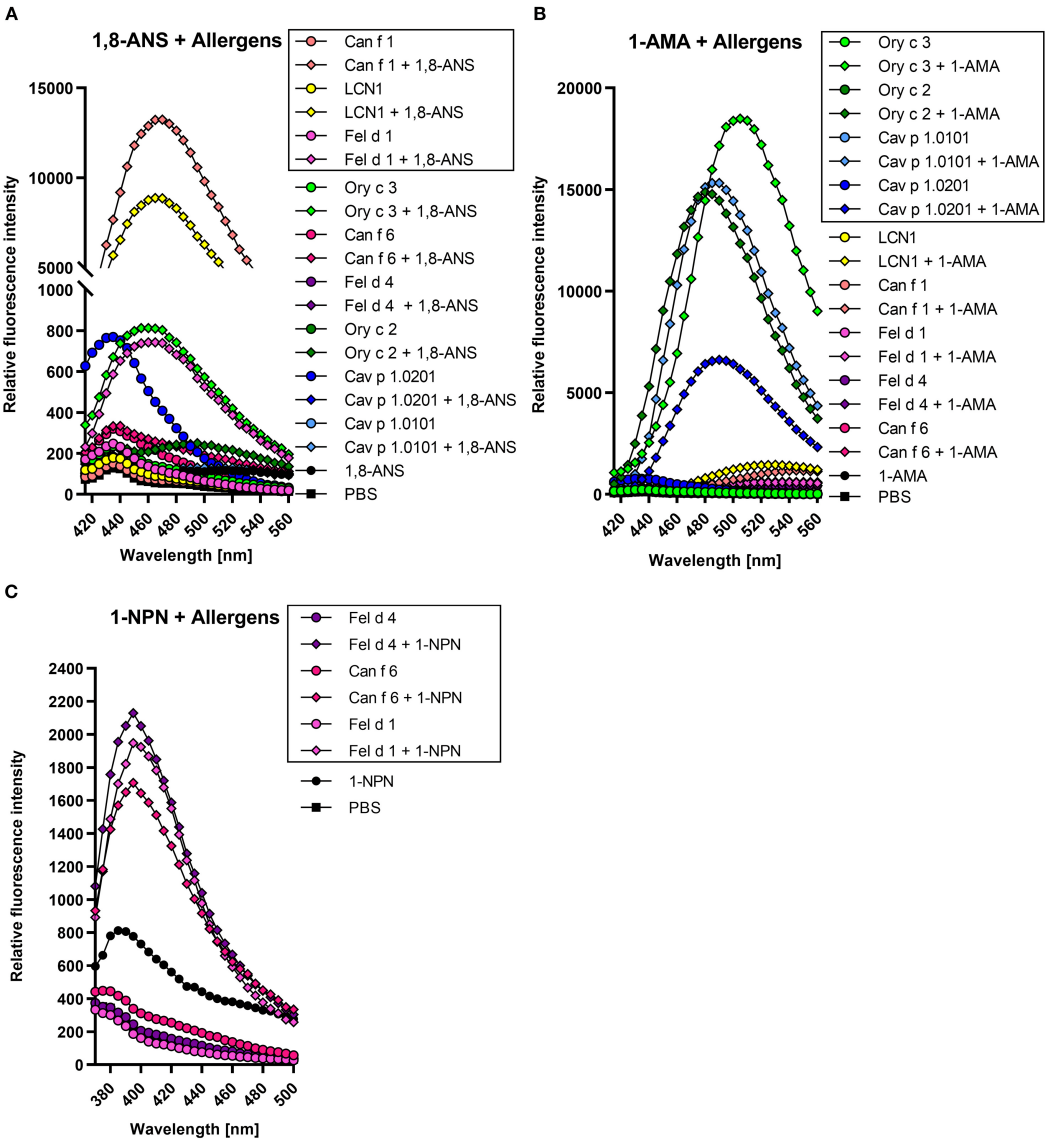
Lipocalin and secretoglobin allergen-fluorochrome complexes emit high fluorescence intensities with protein specific emission maxima. Framed allergen-fluorochrome complexes are chosen for ligand competition assays based on their-signal-to-noise-ratio. Emission spectrum of 5 μM allergen complexed with 5 μM fluorochrome were recorded in duplicates between 420 and 560 nm for **(A)** 1-AMA and **(B)** 1,8-ANS (λ_ex_ = 380 nm) and between 380 and 500 nm for **(C)** 1-NPN (λ_ex_ = 337 nm).

For each allergen, we identified a suitable fluorescence probe by adding equimolar concentrations of either 1-AMA or 1,8-ANS to the recombinant proteins. Fluorescence emission spectra were recorded and are shown in Figure 4. Fluorochrome-allergen pairs exhibiting the greatest increase in fluorescence intensities were chosen for further experiments. Binding of 1,8-ANS to dog allergen Can f 1 and its closely related human LCN1 shifted the emission maximum from 435 to 465 and 470 nm, respectively, and resulted in the most significant increase (>100-fold) of fluorescence intensity compared to other allergens (Figure 4A). 1-AMA was chosen for the guinea pig lipocalin Cav p 1 isoallergens, rabbit lipocalin Ory c 2, and rabbit secretoglobin Ory c 3. Fluorescence intensities increased 20–60-fold upon binding. While the emission maxima shifted from 565 to 485 nm for lipocalin fluorochrome-allergen pairs, the blue shift was less pronounced for the Ory c 3 secretoglobin-1-AMA complex to 505 nm (Figure 4B). Interestingly, the cat derived major allergen Fel d 1, belonging to the same protein family of secretoglobins, only showed negligible interactions with 1-AMA and only an 8-fold fluorescence intensity increase when complexed with 1,8-ANS. Cat and dog lipocalin Fel d 4 and Can f 6 neither reacted with 1-AMA and 1,8-ANS, nor with the fluorescent probe DAUDA (data not shown). 1-NPN paired with Fel d 1, Fel d 4 or Can f 6 resulted in a weak, but sufficient 2–3-fold increase in fluorescent intensities to distinguish fluorochrome and protein background signals from the complexed forms (Figure 4C). Respective K_d_ were calculated after background subtraction (Figure 4B). As ethanol gave a high background with 1-NPN, these experiments were carried out with methanol to reduce the signal-to-noise-ratio.

**Figure 5:**
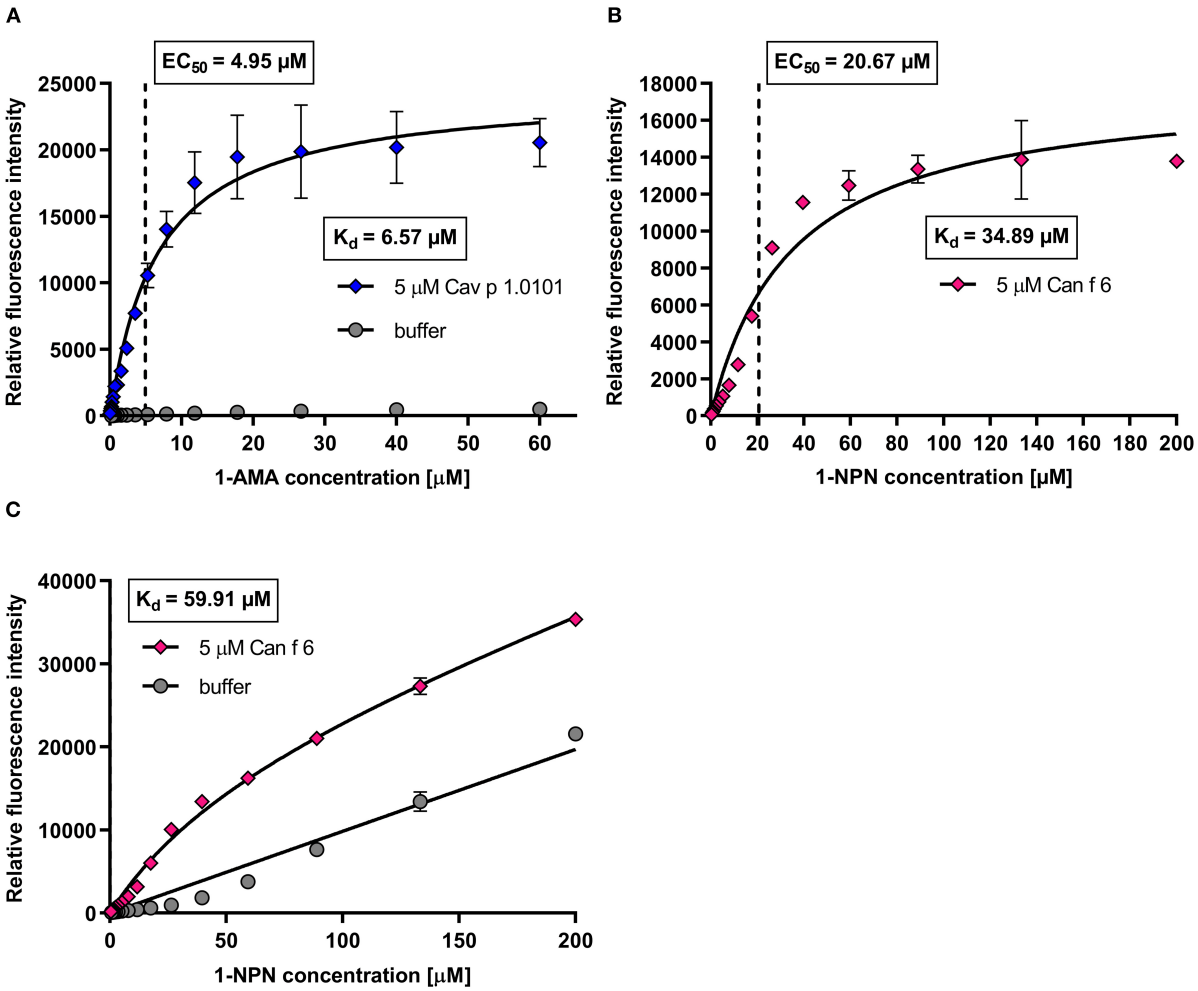
Example of dose-dependent fluorochrome titration curves with guinea pig lipocalin Cav p 1 and dog lipocalin Can f 6. **(A)** Mean fluorescence intensity (λ_ex_ = 380 nm; λ_em_ = 485 nm) of 5 μM Cav p 1.0101 titrated with 1-AMA (0–60 μM) in a 1.5 dilution series. Equilibrium dissociation constants (K_d_) and EC_50_ values were calculated by non-linear regression using a one-site fit specific binding model. Total and non-specific binding are depicted. **(B,C)** Mean fluorescence intensity (λ_ex_ = 337 nm; λ_em_ = 395 nm) of 5 μM Can f 6 titrated with 1-NPN (0–200 μM) in a 1.5 dilution series. K_d_ and EC_50_ values were calculated by one-site fit models of specific binding **(B)** and of total and non-specific binding **(C)**.

We evaluated the binding affinities of the fluorochromes to the allergens by dose-dependent titration curves. Increasing fluorochrome concentrations were added to a fixed protein concentration of 5 μM until saturation (Figure 5). Fluorescence intensities were recorded at specific emission maxima for each fluorochrome-allergen complex, determined using the emission spectra shown in Figure 4. We analyzed the specific binding of fluorochrome-allergen complexes by non-linear regression and calculated equilibrium dissociation constants (K_d_) and EC_50_ values (Table 2). EC_50_ values are equal to fluorochome concentrations in which half of the allergen binding sites are saturated and these concentrations were used in subsequent ligand competition assays. We chose a one-site specific binding model for all allergens, except for human LCN1, where multiple binding site analysis emerged as the model of best fit and two K_d_ values were calculated. Evidence for internal and external binding sites for 1,8-ANS and LCN1 were already reported (58). Since the affinity of 1,8-ANS for LCN1 is significantly lower at the second binding site (13-fold higher K_d_), interferences in the ligand competition assays are unlikely and were neglected.

**Table 2:** Summary of fluorescence-quenching assay parameters and dissociation constants of fluorochromes and the two strongest binding ligands for eight mammalian respiratory allergens and human LCN-1.

**Cluster**	**Allergen (5 μM)**	**Fluorochrome**	**Emission maximum (μM)**	**Fluorochrome dissociation constant (**μ**M)**		**EC**_**50**_ **fluorochrome concentration (**μ**M)**		**Ligand**	**Ligand inhibition constant K**_**i**_ **(**μ**M)**	
				**K_d_**	**95% CI**	**EC_50_**	**95% CI**		**K_i_**	**95% CI**
Cluster 1) Can f 1 and LCN1	Can f 1	1,8-ANS	465	6.8	6.5–7.1	6.4	6–6.9	Arachidonic acid	1.3	0.8–2.4
								Tetradecanoic acid	7.4	4.9–11.5
	Lipocalin-1	1,8-ANS	470	5	2.2–7.8	7.1	5–10.2	Tetradecanoic acid	1.8	0.8–4.2
				(and 64.7)				Arachidonic acid	1.9	1.4–2.5
Cluster 3) Lipocalins: Ory c 2 and Cav p 1 isoallergens	Cav p 1.0101	1-AMA	485	6.6	5.2–8	5	4.4–5.6	Farnesol	0.6	0.4–0.9
								1-Decanol	0.8	0.5–1.3
	Cav p 1.0201	1-AMA	490	13.9	11.6–16.1	9.2	8.2–10.5	Farnesol	0.6	0.4–0.9
								1-Decanol	1.1	0.7–1.8
	Ory c 2	1-AMA	480	5.7	4.5–6.9	4.2	3.7–4.8	Farnesol	1.1	0.9–1.3
								4-Propylphenol	2.6	2.2–3.1
Cluster 3) Secretoglobins: Ory c 3 and Fel d 1	Ory c 3	1-AMA	505	9.0	8–10	6.9	6.4–7.5	1-Dodecanol	0.4	0.3–0.5
								1-Decanol	0.7	0.6–0.9
	Fel d 1	1-NPN	395	35.5	24.8–46.2	21.1	19.9–22.4	Dodecanoic acid	14.4	7.9–27.8
	Fel d 1	1,8-ANS	465	149.4	123.6–175.3	99.8	83.3–119.5	Dodecanoic acid	26.7	19.3–36.8
Cluster 2) Can f 6 and Fel d 4	Fel d 4	1-NPN	395	81.7	46.3–117.1	28	25.8–30.5	2-Isopropylphenol^*^	*20.1*	*9.7*–*42*
								IBMP^*^	*39.2*	*9.7*–*195.8*
	Can f 6	1-NPN	395	34.9	25.3–44.4	20.7	19.6–21.8	IBMP^*^	*49.6*	*17.7*–*176.5*
								Octanamine^*^	*273.4*	*182.3*–*437.8*

* Results in italic have to be interpreted with caution. Suboptimal fluorescence quenching assay parameters led to imprecise Ki values.

Allergens were categorized into three clusters based on similar ligand binding behaviors.

The italic values are indicated to interpreted with caution.

Can f 1, LCN1, Cav p 1.0101, Cav p 1.0201, Ory c 2 and Ory c 3 showed high affinities to 1-AMA and 1,8-ANS in the low micro molar range with K_d_ values between 5.7 and 13.9 μM, whereas Fel d 1, Fel d 4 and Can f 6 showed low affinities to 1-NPN (K_d_ = 34.9–81.7 μM) (Table 2). Non-specific fluorescence signals for 1-AMA and 1,8-ANS allergen-complexes was low (Figure 5A) whereas non-linear regression analysis of total and non-specific binding of 1-NPN titration curves revealed a high background fluorescence, which after subtraction still resulted in a saturated, evaluable curve for allergens with low binding affinity (Figures 5B,C).

### Competitive fluorescence-quenching assays with forty-nine potential ligands revealed characteristic allergen-ligand binding profiles

Whereas, the ligand binding properties of some of the major urinary proteins (MUPs) and odorant binding proteins (OBPs) have been studied, mammalian respiratory lipocalin and secretoglobin allergens are less well characterized and their physiological functions need further investigation. We investigated the ligand-binding behavior of mammalian respiratory allergens from four different species with a panel of forty-nine ligands. A structurally diverse ligand panel was assembled based on similarities to previously reported endogenous and exogenous lipocalin ligands (26, 27, 46, 59, 60). The panel covers several compounds belonging to different chemical classes: fatty alcohols, phenols, fatty acids, terpene derivates, steroids and others. Besides molecules that can be classified as odorants, pheromones or steroids, we also tested lipopolysaccharides and iron catecholate ligands, that mimic bacterial siderophores, to address the antimicrobial function that is attributed to some lipocalins (12, 61).

**Figure 6:**
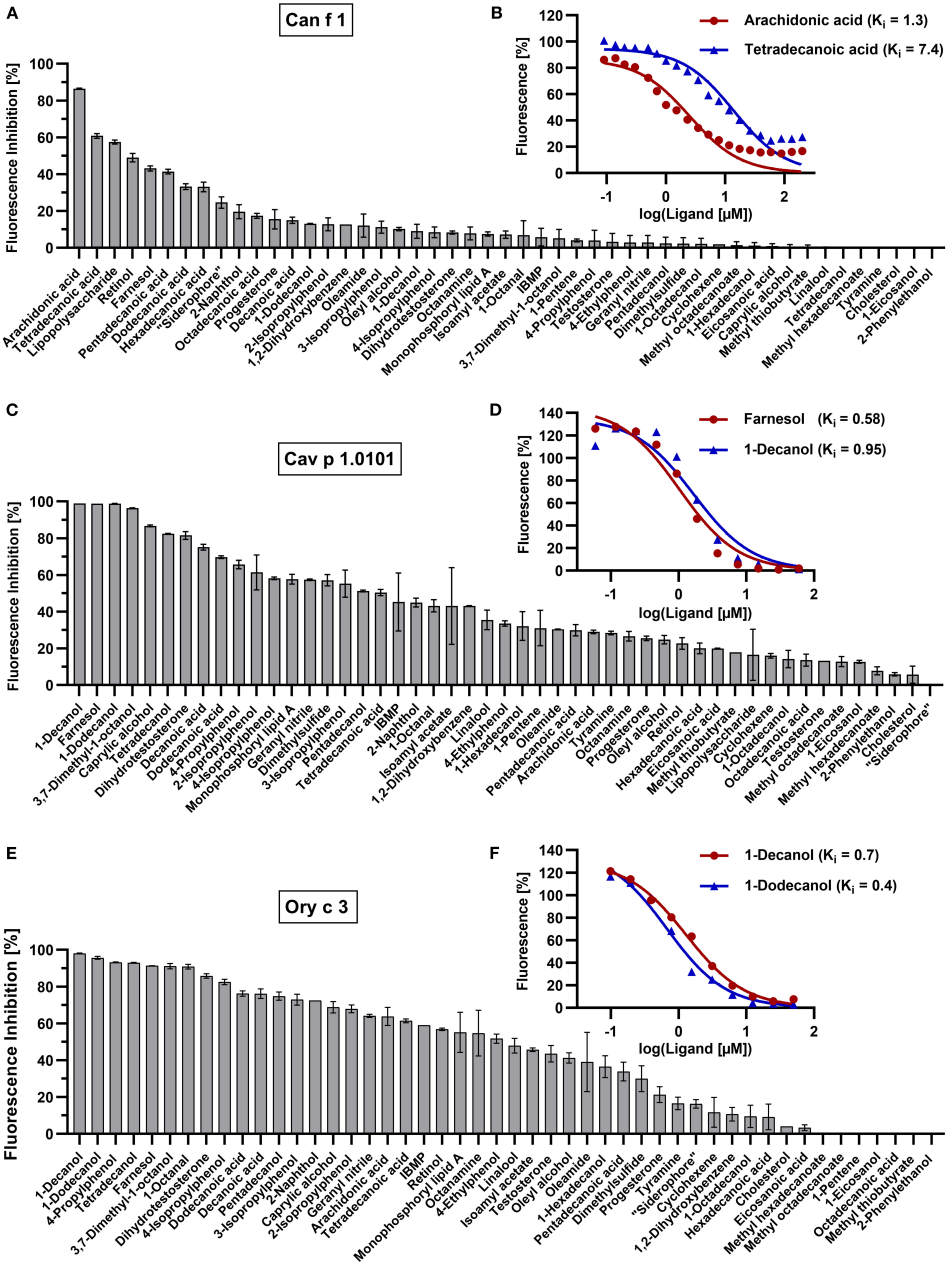
Competition assays with selected allergen-fluorochrome complexes and forty-nine ligands. Mean fluorescence inhibition of 5 μM allergen-fluorochrome complexes by 50 μM competitive ligands in percent (*n* = 2) for **(A)** Can f 1 **(C)** Cav p 1.0101 and **(E)** Ory c 3. Dissociation constants (K_i_) of most effective ligands were calculated by non-linear regression of logarithmized dose-dependent titration curves with **(B)** 0–100 μM arachidonic acid and tetradecanoic acid for Can f 1, **(D)** 0–60 μM farnesol and 1-decanol for Cav p 1.0101, **(F)** 0–50 μM 1-dodecanol and 1-decanol for Ory c 3.

The binding affinities of non-fluorescent ligands is inferred to by their ability to compete and displace the fluorochromes bound to the allergen at a certain concentration. This leads to a measurable decrease in fluorescence intensity that will be expressed as fluorescence inhibition (Figure 6). For a given allergen, the ligand with the strongest inhibition has the highest binding affinity. We used 5 μM of allergen with specific fluorochrome concentrations that were established by titration curves shown in the previous section (EC_50_ values) for each protein. We screened fourty-nine ligands for eight allergens and a human homolog with ligand concentrations of 50 μM and 100 μM for allergen-fluorochrome complexes with low (Can f 1, LCN-1, Cav p 1.0101, Cav p 1.0201, Ory c 2 and Ory c 3) or high K_d_ values (Fel d 1, Fel d 4, Can f 6), respectively. Based on the ligand screening results, the two most effective ligands were chosen for each allergen and titrated to allergen-fluorochrome complexes. Competitive ligand binding was analyzed by non-linear regression and inhibitory dissociation constants (K_i_) were calculated. The results are summarized in Table 2 and selected competition assays with titration curves are depicted in Figure 6. Most allergens displayed a rather promiscuous binding behavior except for the major dog allergen Can f 1, that showed a more specific binding profile in our test panel with arachidonic acid as the strongest binding ligand (K_i_ of 1.3 μM) (Figures 6A,B). Guinea pig and rabbit lipocalins, as well as rabbit secretoglobin Ory c 3 showed strong binding to the previously selected ligands with low dissociation constants between 0.4 and 2.6 μM (Figures 6C–F). By contrast, major cat allergen Fel d 1 showed low binding affinities to the fluorescent probes tested in our experimental set up with K_d_ values of 149.4 μM for 1,8-ANS and 35.5 μM for 1-NPN (Table 2). Still, competition assays with both fluorescent probes resulted in a very similar ligand binding profile and identified dodecanoic acid as one of the strongest binding ligands with K_i_ values of 26.7 and 14.4 μM, respectively. Dodecanoic acid was previously described to be one of the strongest binders to Fel d 1 in a test panel including five fatty acids and nine steroids (27). These findings indicate that fluorescence quenching assays of proteins with high dissociation constants to the fluorescent probes, could still be used as screening tools under the caveat, that exact K_d_ values need further analyses with different techniques. In concordance to this, we were not able to set up optimal fluorescence quenching assay parameters for the cat and dog lipocalins Fel d 4 and Can f 6. Both allergens showed low affinities to the tested fluorescence probes. Competition assays with Can f 6 resulted in inhibition values with a high standard error and dose-dependent titration curves did not reach full saturation leading to unreliable K_i_ values. However, Fel d 4 and Can f 6 exhibited very similar ligand binding profiles.

We observed strong interactions between our ligand panel and the fluorescent probe 1-NPN leading to out of range fluorescence intensities and to distorted inhibition values (data not shown). Therefore, we excluded 17 ligands from our 1-NPN competition assay panel that deviated more than 10% from the 1-NPN blank value. Retinol and progesterone interacting with 1-NPN decreased fluorescence intensities leading to false positive inhibition values, whereas the following compounds (mostly fatty alcohols) strongly increased fluorescence intensities: lipopolysaccharides, farnesol, 1-dodecanol, arachidonic acid, 1-octanal, 2-naphtol, oleamide, oleyl alcohol, pentadecanoic acid, methyl hexadecanoate, methyl octadecanoate, cyclohexene, pentadecanol, tetradecanol, 1-decanol. Abnormal fluorescence emission during competitive ligand-binding experiments with fatty acids and 1-NPN has been previously reported and was associated with concentration dependent ligand micelle formations that could lead to the encapsulation of 1-NPN molecules (62–64).

### Proteins that share higher sequence identities display similar ligand-binding profiles

**Figure 7:**
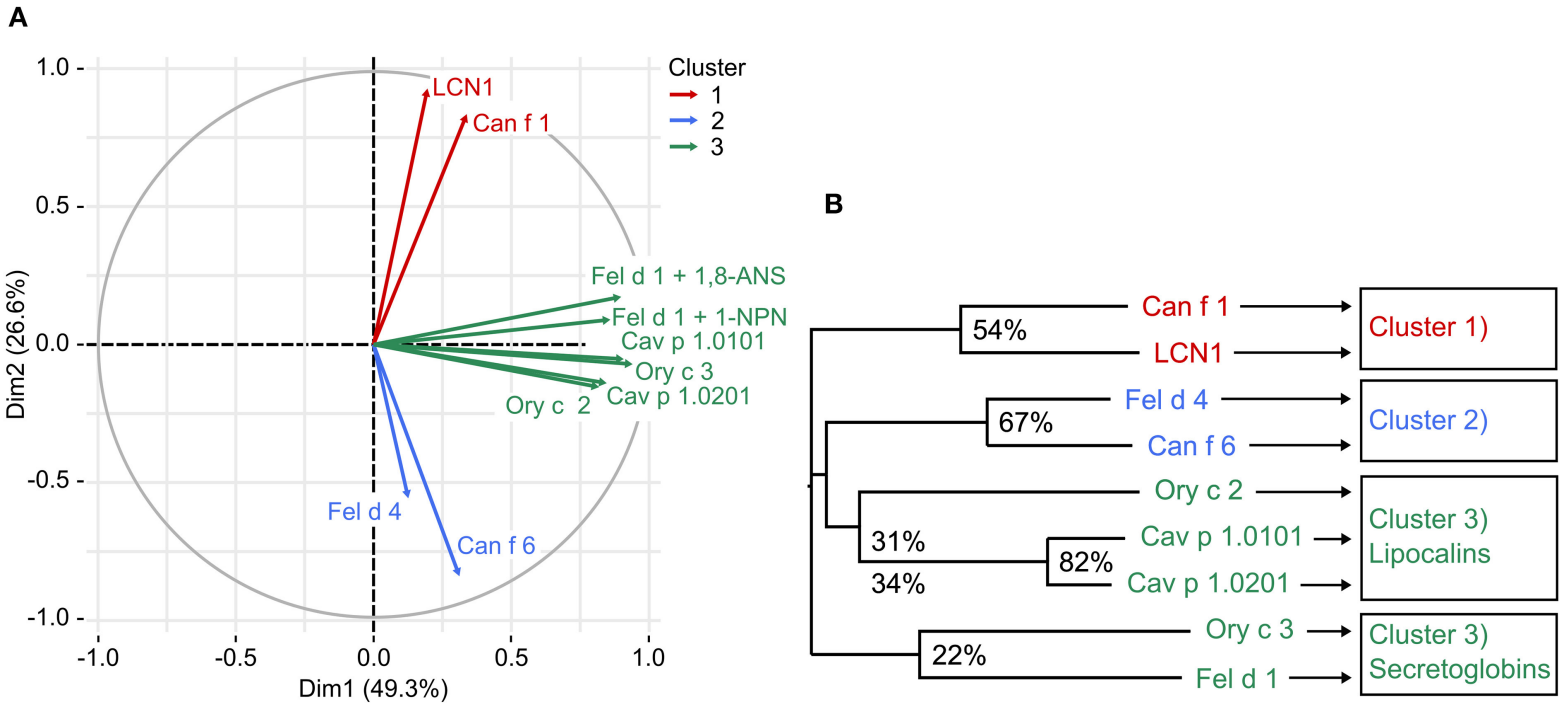
**(A)** Principle component analysis (PCA) identified three clusters of mammalian respiratory allergens with similar ligand binding behavior based on fluorescence inhibition values of forty-nine ligands (thirty-two for 1-NPN). Cluster 3 contains 2 proteins families, lipocalins and secretoglobins, that are unrelated in sequence. **(B)** Phylogenetic tree represents the relationships between eight mammalian respiratory allergens and human LCN1 based on a multiple amino acid sequence alignment. Amino acid sequence identity between cluster members is presented in percentage values.

We observed that allergens sharing higher amino acid sequence identities showed more similarities in their ligand-binding behavior. We compared ligand-binding profiles of all tested allergens and human LCN1 by principle component analysis (PCA). Three clusters were generated (Figure 7). Cluster 3 was further divided into secretoglobin and lipocalin allergens.

Lipocalins are characterized by their highly conserved tertiary structure and generally share a low amino acid sequence identity. Similarities in ligand-binding profiles of some lipocalins, despite their relatively low amino acid sequence identity, point toward the assumption, that only few amino acids are relevant for specific ligand binding. This is especially the case for the cluster formed by Ory c 2 and Cav p 1. A feature of urinary lipocalins in the house mouse is the existence of several isoforms that create an identity signal for individual recognition (65, 66). These isoforms show specific differences in the binding and release of volatile ligands (66, 67). We analyzed the ligand-binding behavior of two lipocalin isoallergens, Cav p 1.0101 and Cav p 1.0201, which are present in the harderian gland, urine and fur of guinea pigs. They share 82.5% amino acid sequence identity. Both isoallergens showed a very similar ligand-binding profile and preferred binding to short chain fatty alcohols between 8 and 15 carbon atoms (C-atoms), as well as to the C15 isoprenoid farnesol, whereas binding to long chain fatty acyls over 17 C-atoms and larger cyclic compounds, such as testosterone or cholesterol, was not observed. Binding affinities of the most effective ligands are highly similar (Table 2). However, the K_d_ value of the fluorescent probe 1-AMA was twice as high for Cav p 1.0201 than for Cav p 1.0101 and slight differences were observed in the rank order of most effective binders, already for the best five ligands (Table 3). Rabbit lipocalin Ory c 2 is present in the same ligand-binding cluster as the guinea pig isoallergens and is further characterized by its high binding affinity to phenols and geranyl nitrile. Rabbit secretoglobin Ory c 3 displayed the most promiscuous binding pattern: seven different ligands showed fluorescence inhibition values over 90% of the 1-AMA-Ory c 3 complex with fatty alcohols being the most prominent ligands, as well as 4-propylphenol and 1-octanal (Figure 6E). Its ligand-binding profile mostly resembles that of Cav p 1.0101. Cat secretoglobin Fel d 1 showed highest binding affinities to saturated straight medium-chain fatty acids and fatty alcohols between 8 and 12 C-atoms. Irrespective of the few identical amino acids shared with Ory c 3 (20%), the rank order of the best ligands of the two secretoglobins exhibit similarities. Major dog allergen Can f 1 clustered together with its human homolog LCN1. Their ligand-binding profiles strongly resembled each other in terms of ligand rank order. However, Can f 1 showed a low affinity to all tested ligands except for arachidonic acid, whereas LCN1 binds a large array of ligands with high affinity as previously reported (12). Compared to other tested allergens, binding of larger molecules such as long chain fatty acids between 14 and 20 C-atoms is favored.

**Table 3:** Mean fluorescence inhibition of 5 μM allergen-fluorochrome complexes by either 50 μM (Can f 1, LCN1, Cav p 1.0101, Cav p 1.0201, Ory c 2, Ory c 3) or 100 μM (Fel d 1, Fel d 4, Can f 6) of the five strongest ligands marked as bold characters.

**Cluster 1) Can f 1 and LCN1**				**Cluster 2) Can f 6 and Fel d 4**			
	**Can f 1**	**LCN1**			**Fel d 4**	**Can f 6**	
Arachidonic acid	**86.4**	**96.7**		2-Isopropylphenol	**99.5**	**104.6**	
Tetradecanoic acid	**60.8**	**91.9**		IBMP	**96.8**	**118.4**	
Lipopolysaccharide	**57.4**	81.2		Octanamine	**94.9**	**148.7**	
Retinol	**49.0**	**84.3**		Testosterone	**92.2**	43.7	
Farnesol	**43.1**	**90.9**		3,7-dimethyl-1-octanol	**89.5**	**100.4**	
Pentadecanoic acid	41.4	**88.7**		3-Isopropylphenol	89.5	**107.0**	
**Cluster 3) Lipocalins: Ory c 2 & Cav p 1 isoallergens**				**Cluster 3) Secretoglobins: Ory c 3 & Fel d 1**			
	**Cav p 1.0101**	**Cav p 1.0201**	**Ory c 2**		**Ory c 3**	**Fel d 1** + **1,8-ANS**	**Fel d 1** + **1-NPN**
1-Decanol	**99.0**	**98.2**	75.1	1-Decanol	**98.0**	**68.4**	–
Farnesol	**98.9**	**95.5**	**96.0**	1-Dodecanol	**95.6**	44.2	–
1-Dodecanol	**98.9**	**97.0**	**84.6**	4-Propylphenol	**93.2**	52.8	**65.3**
3,7-dimethyl-1-octanol	**96.4**	80.3	72.5	Tetradecanol	**92.9**	0.0	–
Caprylic alcohol	**86.7**	82.1	65.3	Farnesol	**91.3**	11.2	–
Tetradecanol	82.4	**97.9**	55.4	3,7-dimethyl-1-octanol	91.1	**58.3**	**85.4**
4-Propylphenol	65.7	57.0	**93.2**	Dodecanoic acid	76.2	**75.2**	**91.7**
Geranyl nitrile	57.4	89.1	**91.2**	Decanoic acid	76.1	**71.2**	**101.0**
3-Isopropylphenol	55.3	75.7	**89.5**	Caprylic alcohol	68.8	**56.5**	49.2
Pentadecanol	51.3	**94.5**	20.8	Octanamine	54.7	37.7	**81.1**

**Figure 8:**
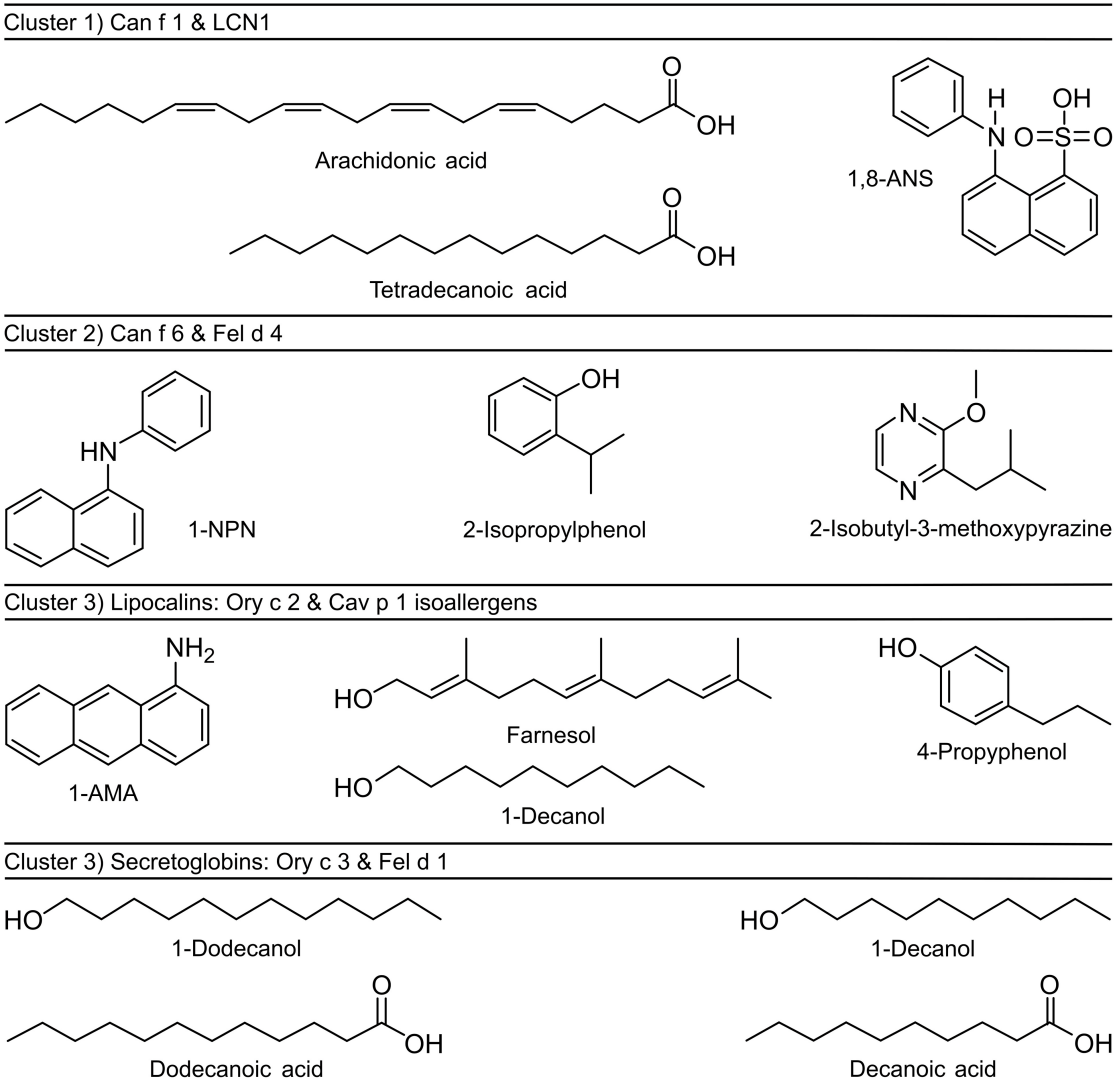
Chemical structures of fluorochromes and strongest binding ligands of mammalian respiratory allergens. Allergens were clustered by similarities in ligand binding profiles and sequence identities.

Cat and dog lipocalin Fel d 4 and Can f 6 (Cluster 2) depict quite a unique binding pattern. The most effective ligands belong to diverse chemical classes but are characterized by small ring structures (8–10 C atoms) (Figure 8). Lipopolysaccharides and monophosphoryl lipid A (MPLA) showed moderate binding based on the ability to displace the fluorescent probes bound to the allergens. Lipopolysaccharide achieved the highest inhibition value with the LCN1-1,8-ANS complex (81%) and MPLA inhibited more than half of the fluorescence emitted by the guinea pig isoallergen- and Ory c 3-fluorochrome complexes.

**Figure 9:**
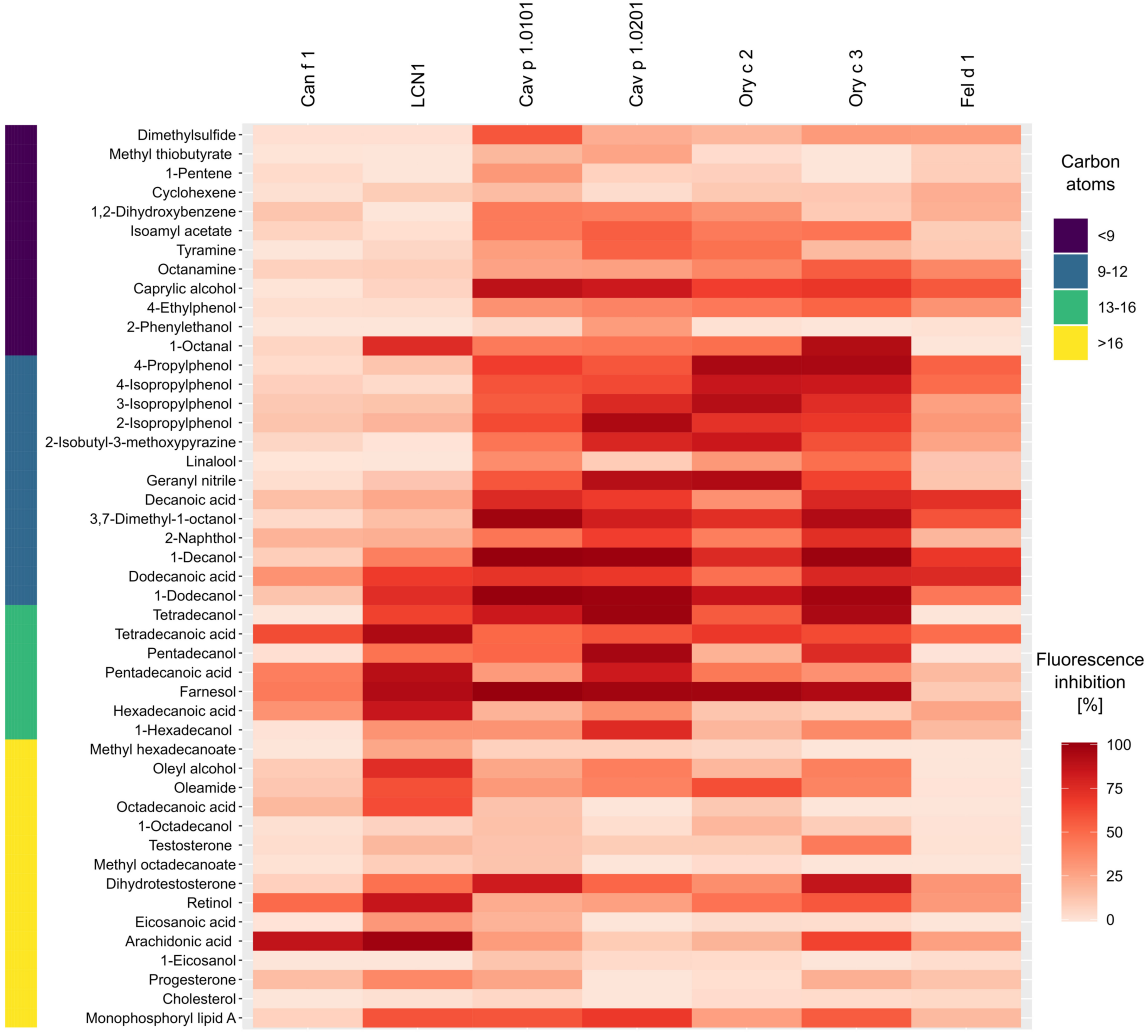
Heat map highlighting universal ligands with high affinities toward four lipocalin and two secretoglobin mammalian respiratory allergens and human LCN1 from dark (high affinity) to faint red (low affinity). Bar on the left represents the number of carbon atoms for each ligand in dark violet < 9, blue 9–12, green 13–16 and yellow > 16. The heat map was created using mean fluorescence inhibition (*n* = 2) of 5 μM allergen-fluorochrome complexes by 50 μM (100 μM for Fel d 1) of forty-seven competitive ligands.

Although we observed specific binding profiles of single allergens and allergen-clusters, certain ligands have emerged as strong universal binders for mammalian respiratory allergens and related homologs in our analysis. Most notably, these were farnesol, 1-dodecanol, 1-decanol, tetradecanoic acid, dodecanoic acid and 3,7-dimethyl-1-octanol (Figure 9). Those ligands are characterized by their size of 9–12 or 14–15 C-atoms, respectively. They are flexible molecules and belong to the families of sesquiterpene alcohols, fatty alcohols and saturated straight chain fatty acids. On the contrary, least effective ligands were large compounds over 17 C-atoms (except for the highly flexible arachidonic acid) such as long chain fatty acyls and steroids with rigid, plate-like ring structures, but also small (<9 C-atoms), highly rigid ring structures.

### Structural comparison of lipocalin and secretoglobin ligand binding cavities

After we established that ligand-binding preferences correlate with the allergen's amino acid sequence, we also investigated their tertiary structure, in particular their binding cavity.

**Figure 10:**
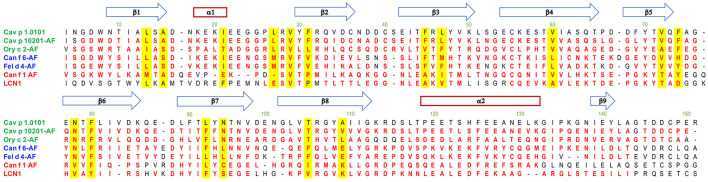
Alignment of lipocalin models with the Cav p 1.0101 structure. Residues structurally aligned with a 2 Å cutoff in PyMol are shown in red. The blue arrows and red rectangle define secondary structures of Cav p 1.0101. Residues with their sidechain contributing to the cavity are highlighted in yellow. Allergen structures with the acronym AF were predicted with AlphaFold v2.0.

A characteristic of lipocalins is their highly conserved tertiary structure despite a generally low amino acid sequence identity between its members. Based on our model protein Cav p 1.0101 we observed that the cavity inside the β-barrel is defined by the side chains of 17 mostly hydrophobic residues (Figures 2D, 10). As expected, the guinea pig isoallergens, which share over 82% amino acid sequence identity and depict a highly similar ligand-binding profile, only show 2 substitutions out of the 17 residues. Although rabbit Ory c 2 shows a similar ligand-binding profile and clusters with the guinea pig isoallergens, it only shares 31–34% amino acid sequence identity with them and has 9 substitutions in the 17 residues that define its binding cavity. However, the substituted amino acids often share comparable properties such as non-polar side chains. Similarly, cluster 1 with dog Can f 1 and human LCN1 share only 7 conserved cavity defining residues, whereas cluster 2 with dog Canf f 6 and cat Fel d 4 have highly similar cavity residues. Particularly noteworthy is the valine residue at position 29, which is highly conserved in all represented lipocalins. All members of cluster 2 and 3 also have an asparagine and phenylalanine at position 78 and 80 in common.

**Figure 11:**
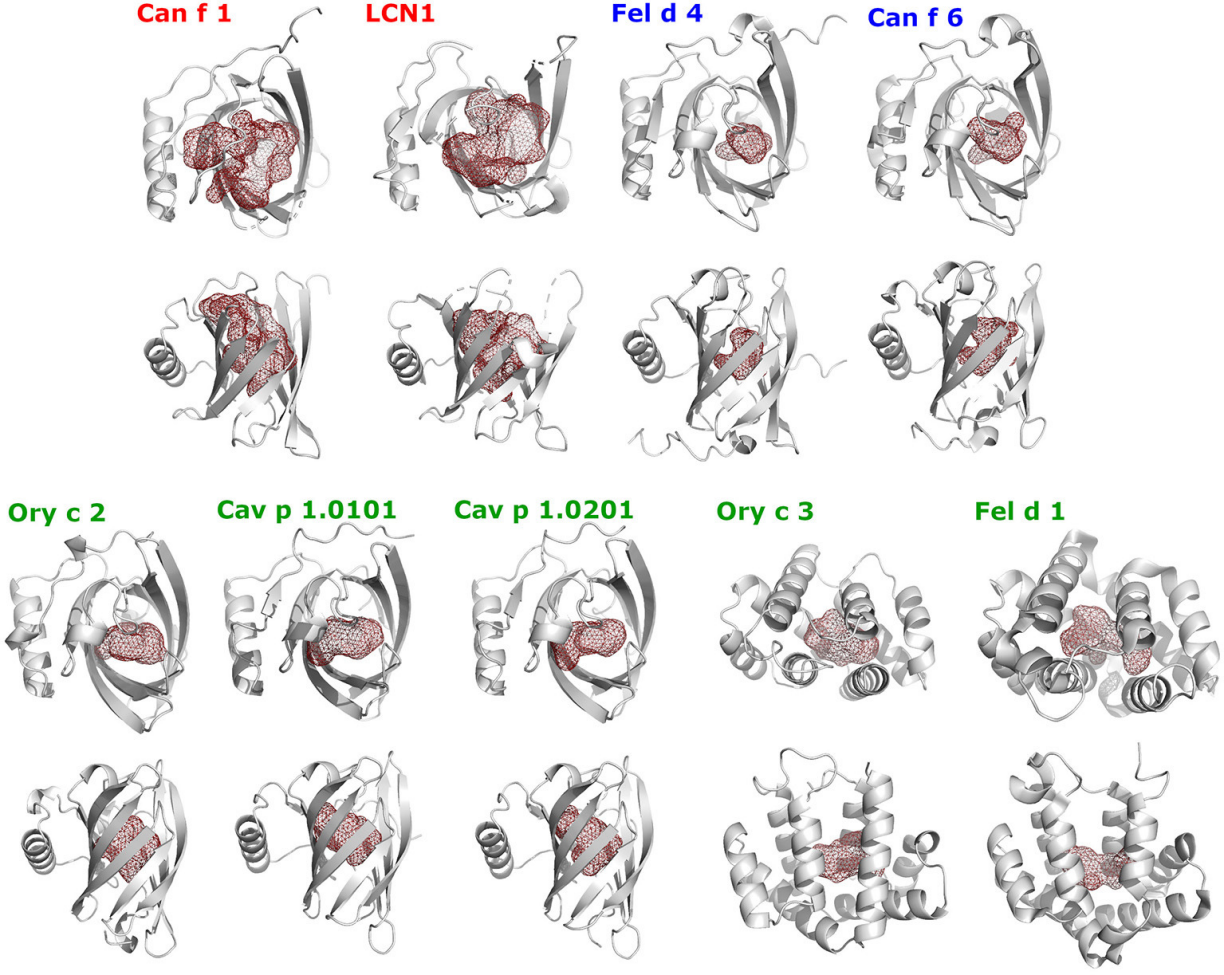
Shapes of ligand-binding cavities of lipocalin and secretoglobin respiratory allergens and human LCN1. Protein structures are shown as gray cartoons in two perpendicular orientations. The ligand-binding cavities were identified using the CASTp webservice and are represented as red colored mesh structures using PyMol.

The cavity shapes and volumes correlate with the ligand-binding clusters and their preferred ligand size (Table 4; Figure 11). Cluster 1, LCN1 in particular, presents the biggest cavities, whereas Cluster 2 with Can f 6 and Fel d 4 have very small cavities. This is in concordance with cluster 1's affinity toward larger molecules over 14 C-atoms, whereas cluster 2 shows higher affinities to small structures under 10 C-atoms.

**Table 4:** Average predicted local distance difference test (pLDDT) of AlphaFold models and cavity volumes computed by CASTp of lipocalin and secretoglobin respiratory allergens and human LCN1.

	**pLDDT**	**Volume cavity [Å^3^]**
**Cluster 1)**		
Can f 1	89.0	203
LCN1	–	394
**Cluster 2)**		
Can f 6	93	35
Fel d 4	91.8	37
**Cluster 3) Lipocalins**		
Cav p 1.0101		109
Cav p 1.0201	95.3	81
Ory c 2	94.2	100
**Cluster 3) Secretoglobins**		
Fel d 1	91.9	59
Ory c 3	95.7	88

In conclusion, the three clusters that were generated based on the ligand-binding preferences of eight allergens and human LCN1, not only reflect their relationship on the primary sequence level, but also in their tertiary structure.

## Discussion

### Physiological implications of ligand binding to allergens

Although a large number of plant and animal allergens have been isolated and characterized in the last decades, the inherent molecular features leading to allergic sensitization remain elusive for the majority of these allergens. In addition to specific physicochemical characteristics such as high stability to environmental denaturation, there is increasing evidence that intrinsic functional or adjuvant properties are leading to allergic sensitization (68). In particular, the binding of small hydrophobic ligands to allergens could be associated with increased thermal and enzymatic stability as well as immune modulation (32).

In the present study, we aimed to characterize the ligand binding profile of eight major respiratory allergens from four furry pets by using a competitive fluorescence quenching assay in a 96-well format. This large-scale screening approach allowed us to compare the affinities of numerous ligands to a specific allergen at one shot, diminishing inter-assay variations. Among the identification of strong binding ligands, we showed that amino acid sequence identity directs ligand-binding specificity and cavity shape of the analyzed recombinant allergens. PCA analysis clearly distinguished the proteins based on their ligand-binding profiles into three clusters. These clusters coincide with distinct subtypes belonging to the lipocalin family. Cluster 1 comprises major dog allergen Can f 1 and it‘s human homolog LCN1, the name giver of the group of the VEG proteins. LCN1 emerged as the most promiscuous protein among lipocalins tested in our set up, with increased affinities for alkyl chains between 14 and 20 C-atoms in length. LCN1 has been subject to several ligand-binding studies before, summarized by Glasgow (12), confirming our findings and thus validating our assay. Although Can f 1 binds to similar structures as LCN1, it specifically binds arachidonic acid with high affinity, followed by tetradecanoic acid. Both fatty acids are components of the phospholipid cell membrane and arachidonic acid can act as an inflammatory mediator. The family of the VEG proteins has not been shown to be clearly involved in chemical communication, except for one study that shows the binding of a pig VEG to the sex steroid progesterone (69). Interestingly, tetradecanoic acid has been shown to be a potent semiochemical, contributing as part of the maternal appeasing pheromone of the sow to attract piglets and has a calming effect at weaning (70). The basic maternal appeasing pheromone composition is similar among mammalian species (71) and components have been shown to bind to lipocalins present in pig maternal fluids (72). Tetradecanoic acid is also present in marking secretions of canids (73). The potential involvement of VEG proteins in chemical signaling needs further investigations. Based on the high sequence similarity of 61% between Can f 1 and cat allergen Fel d 7, it would be of great interest to examine if this VEG allergen depicts a similar ligand-binding profile as its dog homolog.

Cluster 2 comprises lipocalin allergens Can f 6 from dog and Fel d 4 from cat. They are related to salivary proteins (SALs) and to major urinary proteins (MUPs) that are found in urine of rodents, and are proven semiochemicals. SAL1 was purified from boar saliva together with its tightly bound ligands androstenone and androstenol, well-known sex pheromones of the pig (74). SAL1 shows a clear homology with over 60% amino acid sequence identity to a group of major lipocalin allergens derived from horse (Equ c 1), cat (Fel d 4) and dog (Can f 6), that are responsible for a large proportion of clinically relevant IgE cross-reactivity in patients with mammalian respiratory allergies (34, 75). Another allergen belonging to this group is Ory c 4, a rabbit allergen found in seminal fluid that has been speculated to be a semiochemical carrier as well (28, 76). *In vitro* ligand binding assays with native horse allergen Equ c 1 showed affinity of Equ c 1 to small volatile compounds between 9 and 11 C-atoms. Among them emerged 3,7-dimethyl-1-octanol as the best binder, displacing the used fluorescent probe 1-NPN better than its natural ligand oleamide (26). Fel d 4 and Can f 6 also showed a high binding affinity to this compound and especially bound small rigid ring structures as 2-isobutyl-3-methoxypyrazine (IBMP), a model odorant used in several ligand-binding studies, that also binds to MUP (77) and also to SAL1 (78).

Cluster 3 comprises two protein families that are unrelated in sequence and structure but share a similar ligand-binding profile. Even between family members, sequence identity is low (22–31%), excluding the isoallergens among each other. Members are the lipocalin guinea pig Cav p 1 isoallergens and rabbit Ory c 2 and the secretoglobins rabbit Ory c 3 and cat Fel d 1. Guinea pig allergen Cav p 1 isoallergens are found in hair and are expressed in the harderian gland (39). They share over 41% amino acid sequence identity with aphrodisin. This lipocalin is a hamster odorant-binding protein (OBP) that is secreted into the vaginal discharge and is able to elicit copulatory mounting behavior in males by activating their vomeronasal organ (79). Aphrodisin was found to bind a multitude of endogenous ligands, predominantly long chain alcohols as 1-hexadecanol and 1-octadecanol (59). So far, it is not clear, if the aphrodisiac effect is caused by the OBP or its bound ligand, or if a complex of both is essential. Cav p 1 isoallergens and Ory c 2 however, showed strongest binding affinity to the sesquiterpene farnesol and the short chain fatty alcohol 1-decanol. Farnesol is already a known OBP ligand (62, 80), also to insect OBPs (64), where its structural analog (E)-ß-farnesene acts as an alarm pheromone. In vertebrates, OBPs are abundant in the nasal mucosa and have been ascribed with scavenging functions. Either, to remove background odors to facilitate signaling of relevant compounds (18) and remove organic toxins (81), or by binding Quorum sensing molecules such as farnesol, that are essential for bacterial communication, providing OBPs additionally with antimicrobial acitivtiy (80). Farnesol was also identified as a natural ligand of the MUP alpha 2u-globulin in the field rat (82) and was thought to contribute in pheromonal communication.

Similar to the cluster 3 lipocalins, rabbit secretoglobin Ory c 3 shows the highest binding affinities to fatty alcohols as 1-dodecanol, whereas cat secretoglobin Fel d 1 prefers the acidic form dodecanoic acid. Bienboire-Frosini et al. previously reported Fel d 1 to be a semiochemical carrier (27). In a combined *in vitro* and *in silico* approach they also identified dodecanoic acid and additionally androstenone as the strongest binding ligands out of fifteen fatty acids and thirteen steroids tested (27). Dodecanoic acid and tetradecanoic acid are part of the marking fluid composition in large felines (83–85) and seem to be of biological relevance for chemical communication (83). Fel d 1 is closely related to secretoglobins that exist in the mouse (13, 86), also called androgen-binding proteins (ABPs). They play a role in salivary communication (19) and also bind sex steroid hormones (87). Other secretoglobins binding sex steroids and pheromones, respectively, are present in rabbit (88) and pig (89) as well.

The three clusters that were generated by ligand binding preferences of eight allergens in our study also reflect their relationship in amino acid sequence. Especially the ligand binding profiles within the lipocalin family are diverse and cluster into the lipocalin categories of VEGs, SALs/MUPs and OBPs, accordingly. It has been shown previously that distinct lipocalin subtypes with diverse sequence identities interact with specific ligand subsets of different chemical classes, but only within the same species (62, 90). The differences between the lipocalin categories are also reflected in their tertiary structures. The length of the overhanging EF hairpin loop of the β-barrel is likely responsible for restriction and selection of ligands into the binding cavity. LCN-1 has a short EF loop favoring its promiscuous binding behavior (12). Dog lipocalin Can f 6 and cat lipocalin Fel d 4 show a unique conformation at calyx entry compared to other mammalian lipocalins (91). The central β-barrel is closed by the loops. Our findings, that Can f 6, Fel d 4 only bind with high K_d_-values to the fluorochrome 1-NPN support these structural differences compared to other lipocalin allergens. This capping mechanism might be involved in the safe transport of specific ligands and requires a conformational change of the protein. For example, changes in pH can cause changes in loop motion and influence the binding and release of ligands (12). In general, SALs as Can f 6, Fel d 4 and horse allergen Equ c 1 have a rather small binding pocket compared to other lipocalins. They are able to bind small, rigid ring structures as IBMP. *In silico* docking of the closely related boar SAL1 showed that its binding pocket can undergo conformational changes upon binding larger steroid molecules as androstenone or androstenol leading to more favorable van der Waals interactions (92).

### Assay strengths and limitations

A major strength of our study is the systematic screening of a large panel of molecules as potential ligands of major respiratory mammalian allergens. Previous studies have focused on the ligand binding properties of single allergens or of animal lipocalin proteins in the frame of physiological studies. Because of varying assay conditions and different ligands tested, these studies cannot be easily compared to each other and it is not possible to draw overarching conclusions. Here we show for the first time comparative ligand binding profiles of major animal allergens of the lipocalin and secretoglobin families. The 96 well assay format allowed us to screen simultaneously several proteins with up to 49 ligands, limiting the variability of experimental conditions. An advantage of using well-characterized recombinant proteins is their availability and purity, but an inherent disadvantage is the lack of posttranslational modifications such as glycosylation or phosphorylation. Although the 96-well plate screening approach is very convenient, working at a nanomolar scale is also more prone to error and identified ligands would need further investigation by different techniques such as nuclear magnetic resonance (NMR) spectroscopy, isothermal titration calorimetry (ITC), or microscale thermophoresis (MST) to get a more accurate K_d_ value and detailed insight into protein-ligand interactions. Another limitation of our study is that we focused on molecules that had already been reported previously as ligands or are very similar to previously reported ligands. We do not know the natural ligands of the analyzed allergens, although we can infer the structure or chemical class of a potential strong natural ligand, enabling further studies on adjuvant properties of hydrophobic allergen ligands.

### Effects of ligand binding on the allergenic potential of proteins

All major mammalian respiratory allergens known to date are secreted transport proteins with the exception of dog allergen Can f 5, a kallikrein, whose function as a serine protease might explain its sensitizing capacity (93, 94). The sensitization mechanism of lipocalins and secretoglobins is unknown. Among their prominent features as secreted transport proteins that are stability and abundancy, they share the ability to bind small, volatile compounds. Binding of ligands can alter characteristics that contribute to an allergen's sensitizing capacity (32). Especially lipid mediators appear as effective ligands in most studies (95). It is striking that various allergens belonging to different protein families, present in the plant as well as in the animal kingdom, share the ability to interact with lipid ligands such as fatty acids, sphingolipids and sterols (95). It is probable, that this feature is somehow associated with their allergenic potential. More specifically, the binding of fatty acids can affect the allergen‘s structural conformation, thereby enhancing stability, proteolytic resistance and altering its processing (96) or increasing its IgE binding capacity (97). Lipids can act as adjuvants (98) and activate innate immune signaling pathways (99–101). Similarly, binding of bacterial lipopolysaccharides (LPS) to the major secretoglobin allergen Fel d 1 enhances Toll-like receptor 4 signaling (101) and the lipocalin allergen Can f 6 seems to display similar immunomodulatory properties when combined with LPS (101). In the opposite direction, when the lipocalin allergen Bos d 5 (β-lactoglobulin) is loaded with complexed iron or retinoic acid, it acts as immunosuppressor, compared to the form devoid of ligand (46, 102, 103). Ligand binding may also induce the formation of multimeric lipocalin complexes (104). The dimerization of lipocalin allergens and secrectoglobin Fel d 1 likely impacts their allergenic features and stability (105, 106).

Furthermore, ligands can have intrinsic immunomodulatory capacities themselves (32). In our study, we identified a group of ligands exerting relatively high binding affinities to several allergens. This phenomenon can partly be explained by their flexible structures that lead to stronger binding affinities (107). Ligands that are not a protein's physiological ligand still can exhibit high binding affinities. Several of the allergen-binding ligands we identified in our study are used in cosmetics as odorants or surfactants and some of them have been associated with dose-dependent irritations of skin, eyes and respiratory tract. The most prominent promiscuous ligand in our study was farnesol. Farnesol is a contact allergen (108) and is one out of twenty-six fragrances that need to be declared after the European cosmetic regulations (109). Farnesol is included in a routine patch testing series (Fragrance Mix II) in patients with suspected contact allergy against fragrances. One study found a prevalence of 0.4% for contact allergy against farnesol in the general population of five European countries (110). A molecular mechanism of how small contact allergens as farnesol are inducing hypersensitivity reactions was recently proposed (111). Farnesol indirectly activates T-cells by binding to CD1a, an abundant lipid-antigen presenting molecule in human skin. Thereby farnesol buries deeply within CD1a, where it displaces self-lipids and unmasks the CD1a surface (111).

A selection of fatty alcohols and fatty acids with a length between 9 and 14 C-atoms, showed strong binding affinities to several allergens. These compounds, 1-decanol, 1-dodecanol, dodecanoic acid and tetradecanoic acid, are also used in pharmaceutical and cosmetic products as enhancers for skin permeation and drug delivery (112, 113). A prolonged penetration is desirable to hydrate the skin and deliver agents. However, formulations that showed great permeation also showed an increase in skin irritations (114). There is a constant evolution of cosmetic products on the market and new products are developed that use liposomes and lipid nanoparticles as carriers for cosmetic agents (115).

The advent of industrialization is accompanied by an increase in the number of chemicals that we are exposed to. Several of the allergens we tested showed a rather promiscuous binding behavior to a multitude of different ligands, which could be explained by a potential scavenger function. We hypothesize that exogenous ligands, i.e., ligands that are not an allergen's natural ligand, could also bind with high affinities. Concurrently to the enhanced environmental exposure, the prevalence and severity of allergic diseases are on the rise (116, 117). We speculate that, in an era of heavy air pollution and detergent overuse, promiscuous binding transport proteins might be especially vulnerable for interactions with small compound molecules in the environment of the westernized world. Potential ligands could act as irritants and have a disturbing effect on the host‘s immune homeostasis. In addition, the binding of allergens to carriers such as diesel exhaust particles (118, 119) exacerbates allergic symptoms (120, 121). Due to their small and compact structure, the binding sites of lipocalins can easily be modified by few amino acid substitutions, making them valuable tools in the development of biopharmaceuticals (122) and biosensors (123). One of those studies showed that pigOBP with only one amino acid substitution binds polycyclic aromatic hydrocarbons in the low micromolar range (124). The lipocalin alpha 2u-globulin is the major allergen of rat urine. It binds industrial chemicals found in unleaded gas and causes A2U nephropathy (125), a disease unique to male rats where ligand binding prevents the degradation of the protein leading to accumulation in the kidney.

### Conclusion

In conclusion, the systematic screening of major respiratory mammalian lipocalin and secretoglobin allergens with a large panel of potential ligands allowed us to define three clusters of allergens, based on their ligand-binding profiles, which are in accordance with their sequence identity and cavity shape. These clusters also relate to distinct lipocalin sub-families that are associated with a function in chemical communication: VEGs, SALs and MUPs, and OBPs. The secretoglobins Fel d 1 and Ory c 3 form a second group within cluster 3. However, most allergens also showed a rather promiscuous binding to a variety of ligands, notably fatty alcohols and fatty acids that are used in cosmetic products.

Our findings made us aware of the necessity to further investigate the interaction of allergenic transport proteins with a variety of environmental compounds as pollution particles and detergents to gain insight into the allergenic behavior of ligand-binding proteins. When investigating the pathophysiological development of allergies, the allergen molecule itself cannot be seen as a sole entity. Its cargo load and the environmental composition at allergen-host encounter sites has to be accounted for. Therefore, we consider the identification of strong binding ligands, not only as a tool giving us insight into biological functions of transport proteins, but also as a prerequisite to study a molecule's allergenicity.

## Data availability statement

The original contributions presented in the study are included in the article/supplementary materials, further inquiries can be directed to the corresponding author.

## Author contributions

BJ-W, FK, and CH wrote the manuscript. BJ-W performed the experiments and analyzed the data. CH revised the data analysis. CH and AK developed the study concept. FK performed crystallization experiments. KS and SK provided significant groundwork for the conducted experiments. RC provided help with data analysis and graph design. DR performed mass spectrometry experiments. AK, MO, and CB-J provided critical feedback to the manuscript and scientific content. All authors critically revised the manuscript and approved the submitted version.

## Funding

This study was funded with support of the Luxembourg National Research Fund (FNR, PRIDE program grant PRIDE/11012546/NEXTIMMUNE and PRIDE/17/11823097/MICROH) and by institutional funding from Luxembourg Institute of Health.

## Conflict of interest

The authors declare that the research was conducted in the absence of any commercial or financial relationships that could be construed as a potential conflict of interest.

## Publisher's note

All claims expressed in this article are solely those of the authors and do not necessarily represent those of their affiliated organizations, or those of the publisher, the editors and the reviewers. Any product that may be evaluated in this article, or claim that may be made by its manufacturer, is not guaranteed or endorsed by the publisher.

## Abbreviations

1-AMA: 1-Aminoanthracene
ABP: Androgen-binding protein
1,8-ANS: 8-Anilino-1-naphthalensulfonic acid
CD: Circular dichroism
DAUDA: 11-(Dansylamino)undecanoic acid
K_d_: Dissociation constant
IBMP: 2-Isobutyl-3-methoxypyrazine
LCN1: Lipocalin-1
LPS: Lipopolysaccharides
MS: Mass spectrometry
MW: Molecular weight
MUP: Major urinary protein
1-NPN: N-Phenyl-1-naphthylamine
PMF: Peptide mass finger printing
PBS: Phosphate buffered saline
PCA: Principal component analysis
RT: Room temperature
SAL: Salivary protein
SIgE: specific Immunoglobulin E
AC: accession number
VEG: Von Ebner's gland protein.
