# Supplementary material for: Mammalian derived lipocalin and secretoglobin respiratory allergens strongly bind ligands with potentially immune modulating properties
Source: Front Allergy. 2022 Aug 4;3:958711. doi: 10.3389/falgy.2022.958711 (PMC9385959; doi:10.3389/falgy.2022.958711)
Supplement: Supplementary file 1 [file Table_1.DOCX]

Supplementary Material

# Crystallographic structure data of the guinea pig lipocalin allergen Cav p 1.0101

**Supplement Table 1:** Data collection and refinement statistics

|  | **Cav p 1.0101** |
| --- | --- |
| PDB code | 8A0D |
| **Data Collection:** |  |
| Wavelength | 0.978570 |
| Space group | P2_1_2_1_2_1_ |
| a, b, c (Å) | 74.44 122.85 123.36 |
| α, β, γ (°) | 90, 90, 90 |
| Resolution range (Å)^a^ | 47.5 – 3.7 (3.8 – 3.7) |
| Rmerge (%)^a^ | 27.2 (87.7) |
| <I>/<σI> ^a^ | 10.2 (2.7) |
| Completeness (%)^a^ | 99.9 (100) |
| Redundancy ^a^ | 13.3 (12.0) |
| CC 1/2^a^ | 0.992 (0.87) |
|  |  |
| **Refinement:** |  |
| No. of unique reflections | 21414 |
| R work (%) | 25.6 |
| R free (%) | 26.2 |
| No. Atoms |  |
| Protein | 5815 |
| Solvent | 0 |
| RMS deviations from ideal stereochemistry |  |
| Bond lengths (Å) | 0.007 |
| Bond angles (^o^) | 0.92 |
| Mean B factor (Å^2^) | 95.1 |
| Ramachandran plot: |  |
| Favoured region (%) | 93.0 |
| Allowed regions (%) | 6.3 |
| Outlier regions (%) | 0.7 |
| ^a^ Numbers in parenthesis refer to the highest resolution shell. |  |
